# miR-181a-5p Regulates TNF-α and miR-21a-5p Influences Gualynate-Binding Protein 5 and IL-10 Expression in Macrophages Affecting Host Control of *Brucella abortus* Infection

**DOI:** 10.3389/fimmu.2018.01331

**Published:** 2018-06-11

**Authors:** Patrícia P. Corsetti, Leonardo A. de Almeida, André Nicolau Aquime Gonçalves, Marco Túlio R. Gomes, Erika S. Guimarães, João T. Marques, Sergio C. Oliveira

**Affiliations:** ^1^Departamento de Bioquímica e Imunologia, Instituto de Ciências Biológicas, Universidade Federal de Minas Gerais, Belo Horizonte, Brazil; ^2^Departmento de Microbiologia e Imunologia, Universidade Federal de Alfenas, Alfenas, Brazil; ^3^Laboratorio de Sorologia, Microbiologia e Biologia Molecular, Universidade Federal de Santa Catarina, Florianópolis, Brazil; ^4^Instituto Nacional de Ciência e Tecnologia em Doenças Tropicais (INCT-DT), Conselho Nacional de Desenvolvimento Científico e Tecnológico, Ministério de Ciência Tecnologia e Inovação Salvador, Salvador, Brazil

**Keywords:** microRNA, epigenetics, *Brucella abortus*, guanylate-binding protein 5, inflammation, miR-21a-5p

## Abstract

*Brucella abortus* is a Gram-negative intracellular bacterium that causes a worldwide zoonosis termed brucellosis, which is characterized as a debilitating infection with serious clinical manifestations leading to severe complications. In spite of great advances in studies involving host–*B. abortus* interactions, there are many gaps related to *B. abortus* modulation of the host immune response through regulatory mechanisms. Here, we deep sequenced small RNAs from bone marrow-derived macrophages infected with *B. abortus*, identifying 69 microRNAs (miRNAs) that were differentially expressed during infection. We further validated the expression of four upregulated and five downregulated miRNAs during infection *in vitro* that displayed the same profile in spleens from infected mice at 1, 3, or 6 days post-infection. Among these miRNAs, mmu-miR-181a-5p (upregulated) or mmu-miR-21a-5p (downregulated) were selected for further analysis. First, we determined that changes in the expression of both miRNAs induced by infection were dependent on the adaptor molecule MyD88. Furthermore, evaluating putative targets of mmu-miR-181a-5p, we demonstrated this miRNA negatively regulates *TNF-α* expression following *Brucella* infection. By contrast, miR-21a-5p targets included a negative regulator of IL-10, programmed cell death protein 4, and several guanylate-binding proteins (GBPs). As a result, during infection, miR-21a-5p led to upregulation of *IL-10* expression and downregulation of *GBP5* in macrophages infected with *Brucella*. Since GBP5 and IL-10 are important molecules involved in host control of *Brucella* infection, we decided to investigate the role of mmu-miR-21a-5p in bacterial replication in macrophages. We observed that treating macrophages with a mmu-miR-21a-5p mimic enhanced bacterial growth, whereas transfection of its inhibitor reduced *Brucella* load in macrophages. Taken together, the results indicate that downregulation of mmu-miR-21a-5p induced by infection increases GBP5 levels and decreases *IL-10* expression thus contributing to bacterial control in host cells.

## Introduction

Brucellosis is a disease caused by a Gram-negative, facultative intracellular coccobacillus from *Brucella* genus that groups 10 species classified according to host specificity (1). Brucellosis is considered the most widespread zoonosis representing a great public health problem (2, 3). In humans and animals, brucellosis is characterized by a chronic, sometimes lifelong, debilitating infection with serious clinical manifestations leading to severe complications (4). As an intracellular lifestyle bacterium, *Brucella abortus* reaches its replicative niche within phagocytic cells, most prominently macrophages. Despite *B. abortus* is recognized by several innate immune receptors and triggers inflammatory response against this bacterium, it is able to evade killing in phagolysosomes and replicate successively with an endoplasmic reticulum-associated compartment and a modified autophagosome (5, 6). Moreover, we and others demonstrated that *B. abortus* could modulate the immune response through induction of regulatory cytokines such as IL-10 as negative regulation of pro-inflammatory cytokines, suggesting that this interplay between immune responses enables *B. abortus* persistence in the host (7–9). Recently, studies have increasingly reported the involvement of microRNAs (miRNAs) in the regulation of host responses to bacterial pathogens (10). miRNAs are small non-coding RNAs that negatively regulate gene expression by directly binding to the 3′ untranslated region (3′ UTR) of their mRNA targets. Inflammatory and anti-inflammatory responses can induce changes in transcription, processing, or stabilization of mature or precursor miRNA transcripts (11). Several reports have demonstrated the role of host miRNAs during bacterial infection, including *Helicobacter pylori* (12), *Salmonella enterica* (13, 14), *Listeria monocytogenes* (15, 16), *Mycobacterium* species (17–21), or *Francisella tularensis* (22). Those reports used various approaches to determine which miRNAs are differentially expressed during pathogen infection. High-throughput RNA sequencing (RNAseq) allows unbiased analysis of miRNA signatures associated with infection (23). Of note, Zheng et al. (24) described the miRNA expression profile of *Brucella melitensis*-infected RAW264.7 cells by high-throughput sequencing. They observed several differentially expressed miRNAs but did not define putative targets that could be associated with the response to *B. melitensis* infection (24). Here, we describe a panel of miRNAs that are differentially expressed in *B. abortus*-infected macrophages using high-throughput sequencing of small RNA libraries at early times after infection. We further characterize miRNAs whose regulation is MyD88-dependent *in vivo* and *in vitro*. Finally, we show that one of the miRNAs downregulated during infection, miR-21a-5p, affects host control of *B. abortus* infection by negatively regulating guanylate-binding protein (GBP) 5 and inducing *IL-10* expression.

## Materials and Methods

### Ethics Statement

This study was carried out in strict accordance with the Brazilian laws 6638 and 9605 in Animal Experimentation. The protocol was approved by the Committee on the Ethics of Animal Experiments of the Federal University of Minas Gerais (Permit Number: CETEA no. 104/2011).

### Mice, Cell Culture, and Bacteria

MyD88 KO mice were kindly provided by Shizuo Akira from the Osaka University in Japan. The wild-type strain C57BL/6 mice were purchased from the Federal University of Minas Gerais animal facility (UFMG, Belo Horizonte, Brazil). Genetically deficient and control mice were maintained at UFMG and used at 6–8 weeks of age. Bone marrow cells were obtained from femora and tibia of mice and they were derived in bone marrow-derived macrophages (BMDMs) as previously described (25). *B. abortus* virulent strain 2308 was obtained from our own laboratory collection. They were grown in *Brucella* broth medium (BD Pharmingen, San Diego, CA, USA) for 3 days at 37°C without CO_2_.

### Macrophage Infection With *B. abortus*

Bone marrow-derived macrophages were transfected for 24 h with mimics, inhibitors, or scramble controls at a concentration of 5 pmol/well in 24-well plates containing 5 × 10^5^ cells and then infected with virulent *B. abortus* strain 2308 at a multiplicity of infection of 100:1. Bacteria were centrifuged onto macrophages at 400 × *g* for 10 min at 4°C then incubating the cells for 30 min at 37°C under 7% CO_2_. Macrophages were extensively washed with HBSS to remove extracellular bacteria and incubated for an additional 90 min in medium supplemented with 100 µg/mL gentamicin to kill extracellular bacteria. Thereafter, the antibiotic concentration was decreased to 10 µg/mL. At each time point, samples were washed three times with HBSS before processing. To monitor *Brucella* intracellular survival, infected cells were lysed with 0.1% (vol/vol) Triton X-100 in H_2_O and serial dilutions of lysates were rapidly plated onto *Brucella* broth agar plates to count the number of CFU.

### Real-Time RT-PCR for Pro-Inflammatory Cytokine Expression

Bone marrow-derived macrophages were homogenized with TRIzol reagent (Invitrogen) to isolate total RNA. Reverse transcription of 1 µg from total RNA was performed using illustra™ Ready-To-Go RT-PCR Beads (GE Healthcare, Buckinghamshire, UK). Real-time RT-PCR was conducted in a final volume of 10 µL containing the following: SYBR^®^ Green PCR Master Mix (Applied Biosystems, Foster City, CA, USA), cDNA as the PCR template, and 20 µM of primers. The PCR reaction was performed with ABI 7900 Real-Time PCR System (Applied Biosystems, Foster City, CA, USA), using the following cycling parameters: 60°C for 10 min, 95°C for 10 min, 40 cycles of 95°C for 15 s, and 60°C for 1 min, and a dissociation stage of 95°C for 15 s, 60°C for 1 min, 95°C for 15 s, and 60°C for 15 s. Primers were used to amplify a specific 100- to 120-bp fragment corresponding to specific gene targets as described in Table S1 in Supplementary Material. All data are presented as relative expression units compared with 0 h post-infection after normalization to the *β-actin* gene (ΔΔCt = ΔCt treatment − ΔCt 0 h post-infection) (26). PCR measurements were conducted in triplicate. The differences in the relative expression were analyzed by analysis of variance (ANOVA) followed by Tukey’s test (*p* < 0.05 for statistically significant).

### Small RNA Library Preparation and Sequencing

For deep sequencing, total RNA was isolated from control uninfected macrophages or 30 min after *B. abortus* infection using TRIzol reagent (Invitrogen). Total RNA was sent to FASTERIS SA (Plan-les-Ouates, Switzerland) for construction and sequencing of strand-specific small RNA libraries. Small RNAs (15–50 nt) purified from polyacrylamide gel were used to construct libraries using the Illumina^®^ TruSeq^®^ Small RNA Library Prep Kit for Illumina HiSeq 2000 sequencing (Illumina Inc., San Diego, CA, USA).

### Small Non-Coding RNA Bioinformatics Identification

Raw reads from sequencing of the small RNA libraries were processed to remove adapter sequences using the cutadapt tool^1^ and sequencing quality was analyzed using the FastQC tool.^2^ Remaining sequencing reads were collapsed to optimize mapping in the reference genomes as previously described (27). Reads were mapped to *Mus musculus* (GRCm38) genome or *B. melitensis* biovar Abortus (strain 2308) genome using the Bowtie Program.^3^ Using the BedTool Program,^4^ mapped reads were automatic annotated as miRNAs (miRBase version 21), mRNA, snRNA, snoRNA, rRNA (GRCm38.73), or tRNAs.^5^ Reads mapping to each annotated miRNA were counted using an *in house* script developed by our group. Only miRNAs that were present in both libraries were used to evaluate differential expression. Each miRNA was normalized (RPM—reads per million) according to the formula:

$$\text{RPM }({\text{miRNA}}_{i}{\text{, sample}}_{j})=\frac{\text{expression }({\text{miRNA}}_{i}{\text{, sample}}_{j})}{\sum_{k=1}^{n}\text{expression }({\text{miRNA}}_{k}{\text{, sample}}_{j})}\times 1\text{million}$$

where *i* corresponds to a specific miRNA expression and *j* is the specific library. Differentially expressed miRNAs were chosen by following the criteria of number of reads (minimum of 200) and fold-change between control and infected libraries (at least 1.5-fold). A complete list of miRNAs expressed in control and infected macrophages is in Table S2 in Supplementary Material.

### *In Vitro* or *Ex Vivo* Validation of Differentially Expressed miRNA by Real-Time RT-PCR

The expression of selected miRNAs was validated by qPCR. miRNAs were purified from infected or non-infected (NI) BMDMs and from spleens of NI or 3 days intraperitoneally infected C57BL/6 or MyD88 KO mice using miRNeasy mini kit (QIAGEN). Reverse transcription of miRNAs was performed according to the miScript^®^ II RT kit (QIAGEN) protocol. To access mature miRNA expression, we used miScript SYBR^®^ Green PCR kit (QIAGEN) to detect specific amplification in PCR reaction performed with ABI 7900 Real-Time PCR System (Applied Biosystems, Foster City, CA, USA). All data are presented as relative expression units compared with 0 min, 0 h, 0 days, or untreated cells after normalization to the *SNORD61* gene (ΔΔCt = ΔCt treatment − ΔCt 0 min, 0 h, 0 days, or untreated cells). PCR measurements were conducted in triplicate. The differences in the relative expression were analyzed by ANOVA followed by Tukey’s test (*p* < 0.05 for statistically significant).

### Prediction of Putative Targets of Selected miRNAs

Putative targets of nine differentially expressed miRNAs that were validated by RT-qPCR (upregulated: mmu-miR-151-3p, mmu-miR-155-5p, mmu-miR-181a-5p, and mmu-miR-328-3p; and downregulated: mmu-miR-21a-5p, mmu-miR-98-5p, mmu-miR-145a-5p, mmu-miR-146b-5p, and mmu-miR-374b-5p) were obtained from the miRWalk database.^6^ Putative targets were filtered based on the following criteria: binding *p* value ≥0.95, seed fully matched and binding site location of 3′ UTR and CDS. miRNA targets were further analyzed using Reactome V53^7^ to select for candidates associated with an immune function (Table S3 in Supplementary Material).

### *In Vitro* Evaluation of TNF-α, IL-10, and GBP5 Targets for Selected miRNAs

To evaluate putative targets of selected miRNAs, BMDMs were transfected for 24 h with small, chemically modified double-stranded RNAs that mimic endogenous mmu-miR-181a-5p or mmu-miR-21a-5p (mimics) to enable miRNAs functional analysis by upregulation of their activities. On the other hand, BMDMs were transfected with small, chemically modified single-stranded RNA molecules designed to specifically bind to and inhibit endogenous mmu-miR181a-5p or mmu-miR-21a-5p (inhibitors) and enable miRNA functional analysis by downregulation of miRNA activity (mirVana™ miRNA mimics or inhibitors—Thermo Fischer Scientific, MA, USA). Non-transfected cells or cells transfected with scramble controls (Thermo Fischer Scientific, MA, USA) were used as negative controls. After transfection, cells were infected with *B. abortus*, as described above, and the total RNA was extracted and reverse transcribed in cDNA from mRNA or miRNA as described above.

### Immunoblotting

Bone marrow-derived macrophages transfected with mmu-miR-21a-5p mimic or inhibitor were lysed with M-PER™ Mammalian Protein Extraction Reagent (Thermo Fisher Scientific) supplemented with phosphatase and protease inhibitors (Roche). Equal amounts of proteins were separated on 12% SDS-PAGE gels and transferred to nitrocellulose membranes (Amersham Biosciences, Uppsala, Sweden). Membranes were blocked for 1 h in TBS with 0.1% Tween-20 containing 5% nonfat dry milk and incubated overnight with primary antibodies [anti-GBP5 dilution 1:500 (ProteinTech, Chicago, IL, USA) or β-actin (dilution 1:5,000, Cell Signaling Technology, Danvers, MA, USA)] at 4°C. Membranes were incubated with horseradish peroxidase-conjugated secondary antibody (dilution 1:1,000) and Luminol chemiluminescent HRP substrate (Millipore, Billerica, MA, USA) was used for antibody detection. Densitometry analysis was performed using ImageQuant TL Software (GE Healthcare, Buckinghamshire, UK), and band intensities were normalized to β-actin.

### Statistical Analysis

All experiments (except RNAseq) were repeated at least twice with similar results and figures show data from one representative experiment. The number of replicates for each experiment is mentioned in each specific figure legend. Graphs and data analysis were performed using GraphPad Prism 5 (GraphPad Software), using one-way ANOVA or two-way ANOVA (Bonferroni *post hoc* test).

## Results

### High Pro-Inflammatory Cytokine Gene Expression Occurs in the Initial Phase of Macrophage Infection With *B. abortus*

**Figure 1:**
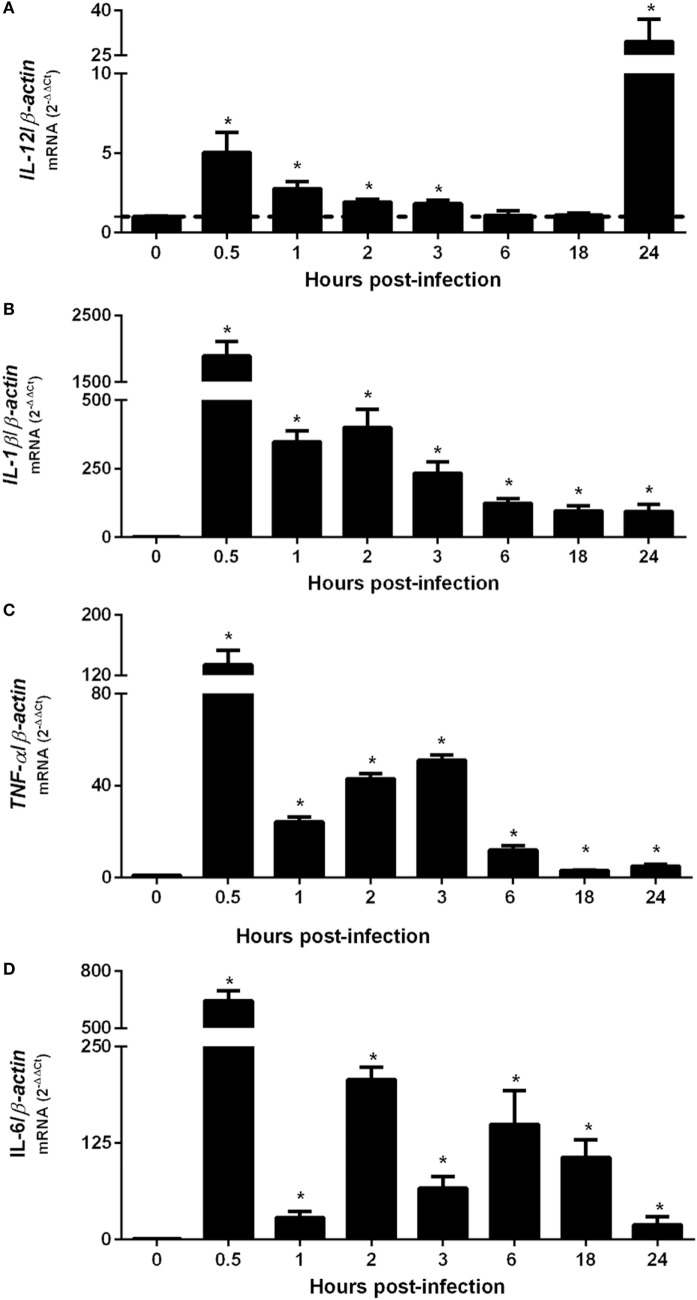
Upregulation of pro-inflammatory cytokine genes occurs 30 min after *Brucella abortus* infection of macrophages. Bone marrow-derived macrophages from C57BL/6 mice were obtained and infected with *B. abortus* strain 2308 for 0.5, 1, 2, 3, 6, 18, or 24 h. Total RNA were extracted and cDNA for IL-12 **(A)**, IL-1β **(B)**, TNF-α **(C)**, or IL-6 **(D)** were assessed by real-time PCR to determine the level of differential expression compared with non-infected (NI) cells (0 h post-infection). The results were normalized to β-actin. Error bars represent the mean ± SD. Similar results were obtained in three independent experiments. Statistically significant differences of gene expression from infected compared with NI cells are denoted by an asterisk (*p* < 0.05).

To evaluate the peak of cytokine gene expression by BMDMs after *B. abortus* infection, total RNA from intracellular-infected cells were transcribed into cDNA and *IL-12, TNF-α, IL-1β*, or *IL-6* transcripts were accessed at different time-points by real-time PCR (Figure 1). The results showed an upregulation of investigated pro-inflammatory cytokines 30 min after BMDM infection with *B. abortus*. *IL-12* expression demonstrated a peak at 30 min and 24 h post-infection (Figure 1A). However, at 24 h post-infection, the levels of detected *IL-12* transcripts were much greater compared with the other cytokines tested. *IL-1β* (Figure 1B), *TNF-α* (Figure 1C), or *IL-6* (Figure 1D) present similar patterns of differential expression with a peak at 30 min post-infection when compared with NI cells. These findings demonstrate that *B. abortus* induces pro-inflammatory cytokine expression in BMDMs at 30 min after infection.

### High-Throughput Sequencing of Small RNA Libraries Identifies Differential Expression of miRNAs During Macrophage Infection With *B. abortus*

**Figure 2:**
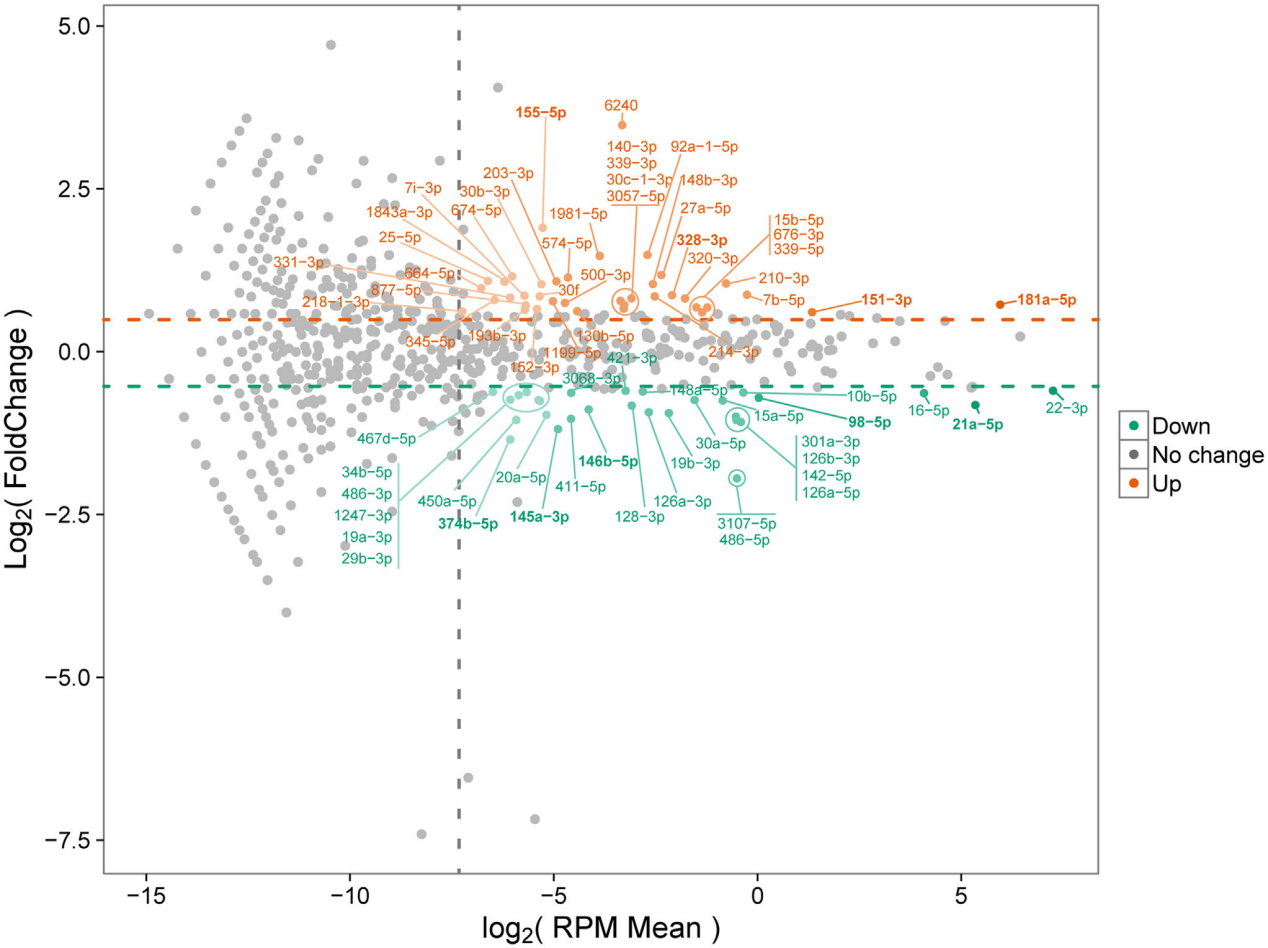
MA plot of differentially expressed microRNAs (miRNAs). Differential miRNA expression between control and infected macrophages is shown. Upregulated and downregulated miRNAs in infected samples compared with controls are indicated. Only miRNAs with at least 200 reads in each sample were considered. Orange and green circles represent upregulated and downregulated miRNAs, respectively. Horizontal dashed lines represent the upper and lower limits of at least 1.5 fold-change. Vertical dashed line shows the normalized miRNA expression corresponding to 200 reads per sample.

**Figure 3:**
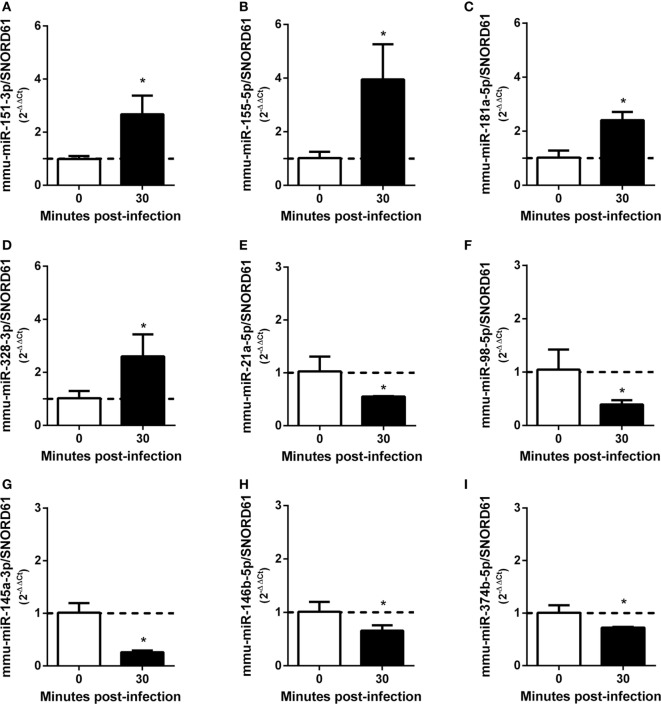
MicroRNAs (miRNAs) differentially expressed during *Brucella abortus* infection in macrophages identified by RNA sequencing were validated by real-time PCR. Four miRNAs were validated by real-time PCR as upregulated: **(A)** mmu-miR-151-3p, **(B)** mmu-miR-155-5p, **(C)** mmu-miR-181a-5p, and **(D)** mmu-miR-328-3p. By contrast, five miRNAs were validated as downregulated: **(E)** mmu-miR-21a-5p, **(F)** mmu-miR-98-5p, **(G)** mmu-miR-145a-3p, **(H)** mmu-miR-146b-5p, and **(I)** mmu-miR-374b-5p. miRNAs expression was assessed by real-time PCR and were normalized to *SNOR61*. Error bars represent the mean ± SD. Similar results were obtained in three independent experiments. Statistically significant differences of miRNAs expression after 30 min bone marrow-derived macrophage infection compared with non-infected cells are denoted by an asterisk (*p* < 0.05).

As demonstrated above that BMDMs trigger upregulation of pro-inflammatory cytokines at 30 min after *B. abortus* infection, we chose this time point to prepare small RNA libraries for high-throughput sequencing. RNAseq revealed that more than 80% of sequenced reads corresponded to 18–26 nt small RNAs with a peak at 22 nt, in both libraries (Table S4 in Supplementary Material). These results indicate that both small RNA libraries were enriched for the expected size of mammalian miRNAs. Small RNAs were mapped to mouse (*M. musculus*—GRCm38) and *B. abortus* (strain 2308) reference genomes. In the small RNA library from infected macrophages, we observed that 92.02% of all mapped reads belonged to the *M. musculus* genome, while 7.43% originated from *B. abortus* (Table S5 in Supplementary Material). In the library from control NI cells, 99.95% of mapped reads originated from the *M. musculus* genome. In the library from control macrophages, we identified 819 miRNAs comparable to 800 detected in *B. abortus*-infected cells. In total, 745 miRNAs were detected in both libraries (complete list is shown in Table S2 in Supplementary Material). Less than 10% of mapped reads did not correspond to miRNAs and corresponded to mRNA, snRNA, snoRNA, rRNA, or tRNA. Therefore, vast majority of small RNAs detected in our samples were miRNAs. We next concentrated our analysis on differentially expressed miRNAs. We observed that, in total, 69 miRNAs had at least 1.5 fold-change in expression comparing control and *Brucella*-infected macrophages and at least 200 reads in each sample (Figure 2; Table S2 in Supplementary Material). RNAseq results were based on a single sequencing experiment to have a broad picture of the host small RNA response to infection. For further validation, we chose four upregulated (mmu-miR-151-3p, mmu-miR-155-5p, mmu-miR-181a-5p, and mmu-miR-328-3p) and five downregulated (mmu-miR-21a-5p, mmu-miR-98-5p, mmu-miR-145a-5p, mmu-miR-146b-5p, and mmu-miR-374b-5p) miRNAs (Table S6 in Supplementary Material) in infected samples by real-time PCR in macrophages. These results corroborated the differential expression of all nine miRNAs in infected macrophages *in vitro* (Figure 3).

**Figure 4:**
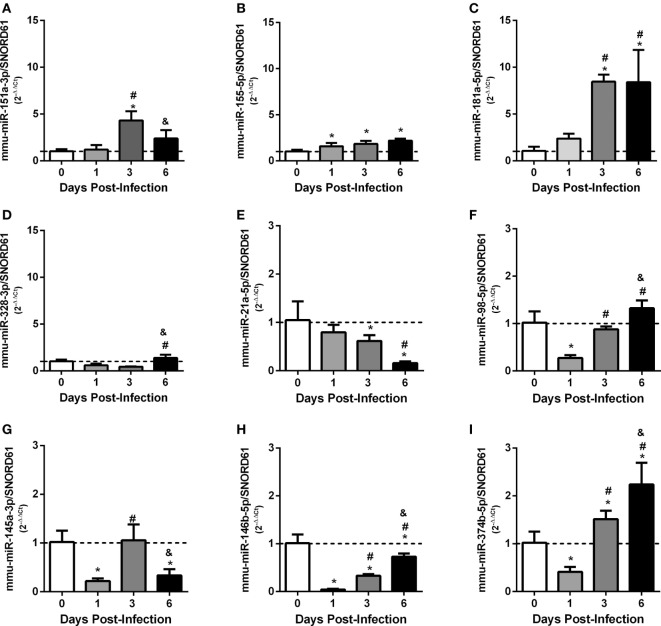
*Ex vivo* analysis of differentially expressed validated microRNAs (miRNAs) in mouse spleens. C57BL/6 mice were infected intraperitoneally at 1, 3, or 6 days post-infection, and the relative expression of miRNAs: **(A)** mmu-miR-151-3p, **(B)** mmu-miR-155-5p, **(C)** mmu-miR-181a-5p, **(D)** mmu-miR-328-3p, **(E)** mmu-miR-21a-5p, **(F)** mmu-miR-98-5p, **(G)** mmu-miR-145a-3p, **(H)** mmu-miR-146b-5p, and **(I)** mmu-miR-374b-5p were evaluated in mouse spleens. miRNAs expression were assessed by real-time PCR and were normalized to *SNOR61*. Error bars represent the mean ± SD. Similar results were obtained in two independent experiments. Statistically significant differences of miRNAs expression from infected mice compared with non-infected mice (0) are denoted by an asterisk (*p* < 0.05), # (*p* < 0.05) represents statistically significant differences compared with 1 day post-infection, and & represents statistically significant differences compared with 3 day post-infection with *B. abortus*.

To analyze the expression of these miRNAs *in vivo*, C57BL/6 mice were infected intraperitoneally with *Brucella*, and the relative expression of miRNAs were evaluated in mouse spleens at 1, 3, or 6 days post-infection. Consistent with *in vitro* results, we observed that all upregulated or downregulated miRNAs presented this profile in at least one time point of infection analyzed (Figures 4A–I).

### MyD88 Plays an Important Role in Regulating the Expression of miRNAs During *B. abortus* Infection

**Figure 5:**
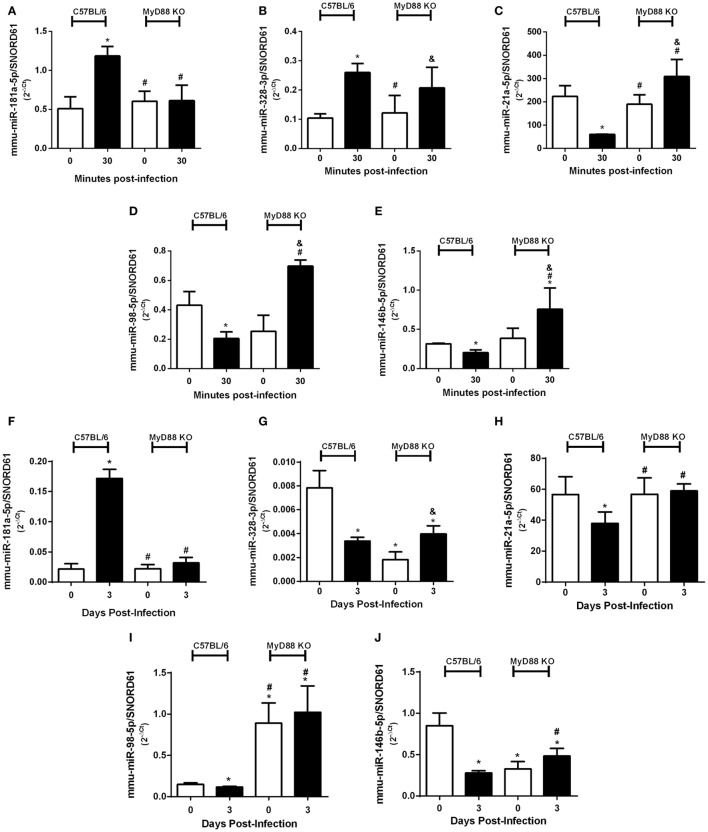
MyD88 plays an important role in differential expression of microRNAs (miRNAs). Differential expression of validated upregulated miRNAs **(A)** mmu-miR-181a-5p and **(B)** mmu-miR-328-3p or validated downregulated miRNAs **(C)** mmu-miR-21a-5p, **(D)** mmu-miR-98-5p, and **(E)** mmu-miR-146b-5p were assessed by real-time PCR and normalized to *SNORD61* in bone marrow-derived macrophages from C57BL/6 and MyD88 KO mice. In addition, the role of MyD88 in miRNA regulation was also observed *ex vivo*. Validated upregulated miRNAs **(F)** mmu-miR-181a-5p and **(G)** mmu-miR-328-3p or validated downregulated miRNAs **(H)** mmu-miR-21a-5p, **(I)** mmu-miR-98-5p, and **(J)** mmu-miR-146b-5p were also assessed by real-time PCR in spleens from C57BL/6 and MyD88 KO mice. Error bars represent the mean ± SD. Similar results were obtained in three independent experiments. Statistically significant differences of miRNAs expression from C57BL/6 infection compared with non-infected (NI) cells are denoted by an asterisk (*p* < 0.05), # compared with C57BL/6 infected (*p* < 0.05), and & compared with NI MyD88 KO cells (*p* < 0.05).

Since innate immunity is the first line of host immune defense against bacterial pathogens and our group has previously demonstrated the important role of MyD88 adaptor molecule during *B. abortus* infection (25), we evaluated the influence of MyD88 during differential expression of miRNAs upregulated (mmu-miR-181a-5p and mmu-miR-328-3p) or downregulated (mmu-miR-21a-5p, mmu-miR-98-5p, and mmu-miR-146b-5p) by infection. We observed a dependence of MyD88 in upregulation of mmu-miR-181a-5p (Figure 5A), while it was not observed differences of upregulation of mmu-miR-328-3p in the absence of MyD88 (Figure 5B). On the other hand, we observed a dependence of MyD88 in the downregulation of all miRNAs we tested during *B. abortus* infection in macrophages (Figures 5C–E). We next evaluated the expression of selected miRNAs in spleens from MyD88 KO compared with wild-type mice (Figures 5F–J). Among upregulated miRNAs, only mmu-miR-181a-5p showed the same profile of MyD88 regulation *in vitro* and *ex vivo* (Figure 5F). Regarding downregulated miRNAs, the dependence on MyD88 was similar *in vitro* as well as *ex vivo*, highlighting the importance of this adaptor molecule in regulating miRNA expression (Figures 5H–J).

### miR-181a-5p and miR-21a-5p Regulate Important Immune Pathways During *B. abortus* Infection

**Figure 6:**
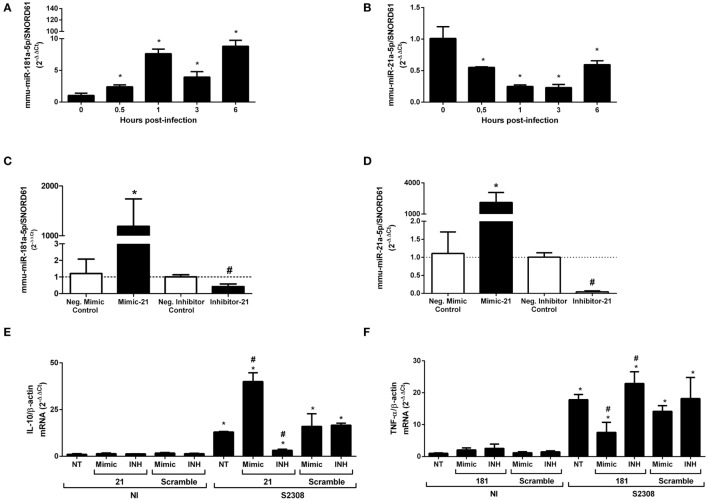
TNF-α expression is modulated by mmu-miR-181a-5p, while IL-10 gene expression is regulated by mmu-miR-21a-5p during *Brucella abortus* infection. First, we determined time dependent of mmu-miR-181a-5p **(A)** or mmu-miR-21a-5p **(B)** expression during *B. abortus* infection in bone marrow-derived macrophages (BMDMs). miRNAs expression was assessed by real-time PCR and was normalized to *SNOR61*. Error bars represent the mean ± SD. Similar results were obtained in two independent experiments. Statistically significant differences of miRNAs expression from infected BMDM compared with non-infected (NI) cells are denoted by an asterisk (*p* < 0.05). Second, mimics and inhibitors transfection for mmu-miR-181a-5p **(C)** or mmu-miR-21a-5p **(D)** showed increase or inhibition of specific miRNAs in BMDMs. Error bars represent the mean ± SD. Similar results were obtained in two independent experiments. Statistically significant differences of miRNAs availability from BMDM transfected with mimic to negative control to mimic (scramble) are denoted by an asterisk (*p* < 0.05), and # (*p* < 0.05) represents statistically significant differences between BMDMs transfected with anti-miR to negative control to inhibitors (scramble). Finally, BMDMs transfected with mimic or inhibitor for mmu-miR-21a-5p was infected with *B. abortus* and the level of *IL-10* mRNA was measured by real-time PCR **(E)**. BMDMs transfected with mimic or inhibitor for mmu-miR-181a-5p was infected with *B. abortus* and the level of TNF-α mRNA was determined by qPCR **(F)**. The results were normalized to *β-actin*. Error bars represent the mean ± SD. Similar results were obtained in two independent experiments. Statistically significant differences of gene expression from infected cells to non-transfected (NT)/NI cells are denoted by an asterisk (*p* < 0.05), and # (*p* < 0.05) represents statistically significant differences with NT/infected (S2308) cells.

mmu-miR-181a-5p and mmu-miR-21a-5p showed differential expression during *B. abortus* infection *in vivo* and *in vitro* in a MyD88-dependent manner. Furthermore, these were the most expressed miRNAs in macrophages that showed differential expression (Figure 2). Therefore, we chose these miRNAs for further studies. First, we analyzed the kinetics of expression for mmu-miR-181a-5p or mmu-miR-21a-5p during *B. abortus* infection in macrophages. As demonstrated in Figures 6A,B, both miRNAs were differentially expressed throughout the time course of infection. Expression of mmu-miR-181a-5p increased during all time-points evaluated when compared with 0 h (Figure 6A). Nevertheless, we observed a slight reduction in mmu-miR-181a-5p expression at 3 h when compared with 1 or 6 h post-infection. We also observed that mmu-miR-21a-5p was downregulated during the first 6 h post-infection (Figure 6B). To characterize the role of these miRNAs during *B. abortus* infection, we transfected macrophages with miRNA mimics or inhibitors for mmu-miR-181a-5p or mmu-miR-21a-5p. We specifically observed reduction of miRNA expression by the inhibitor or increased levels by the mimic (Figures 6C,D).

Each of these miRNAs, mmu-miR-181a-5p and mmu-miR-21a-5p, could regulate several mRNA targets that could affect the host immune responses to *B. abortus* (Table S3 in Supplementary Material). mmu-miR-181a-5p was reported to be important in the regulation of NF-κB activation and TNF-α production (28). More recently, Luo et al. (29) have demonstrated that *Brucella suis* upregulated miR-181a that correlated with decreased TNF-α in Raw264.7 macrophage cell line. To further investigate whether miR-181a-5p can influence TNF-α expression in BMDMs during *B. abortus* infection, we transfected macrophages with the specific mimic or inhibitor for mmu-miR-181a-5p before infection. As observed in Figure 6F when the miR-181a-5p mimic was used, there was a decrease in *TNF-α* transcripts in *B. abortus*-infected macrophages. By contrast, when the miR-181a-5p inhibitor was transfected in BMDMs, we observed an increase in *TNF-α* mRNA levels. These data suggest that upregulation of miR-181a-5p prevents further increase in TNF-α levels during *B. abortus* infection.

One well defined target of miR-21a-5p is programmed cell death protein 4 (PDCD4), which is a pro-inflammatory protein that suppresses IL-10 expression (30). To better understand if the downregulation of miR-21a-5p observed during *B. abortus* infection could modulate *IL-10* mRNA in BMDMs, we used specific miRNA mimics or inhibitors. We observed that the miR-21a-5p mimic induced higher levels of *IL-10* mRNA in infected macrophages while its inhibitor reduced *IL-10* transcripts compared with controls (Figure 6E). These results suggest that the reduction of mmu-miR-21a-5p during *B. abortus* infection helps increase *IL-10* mRNA levels by inhibiting PDCD4 in macrophages.

### miR-21a-5p Regulates GBP5 Expression and Partially Influences Intracellular *B. abortus* Growth

**Figure 7:**
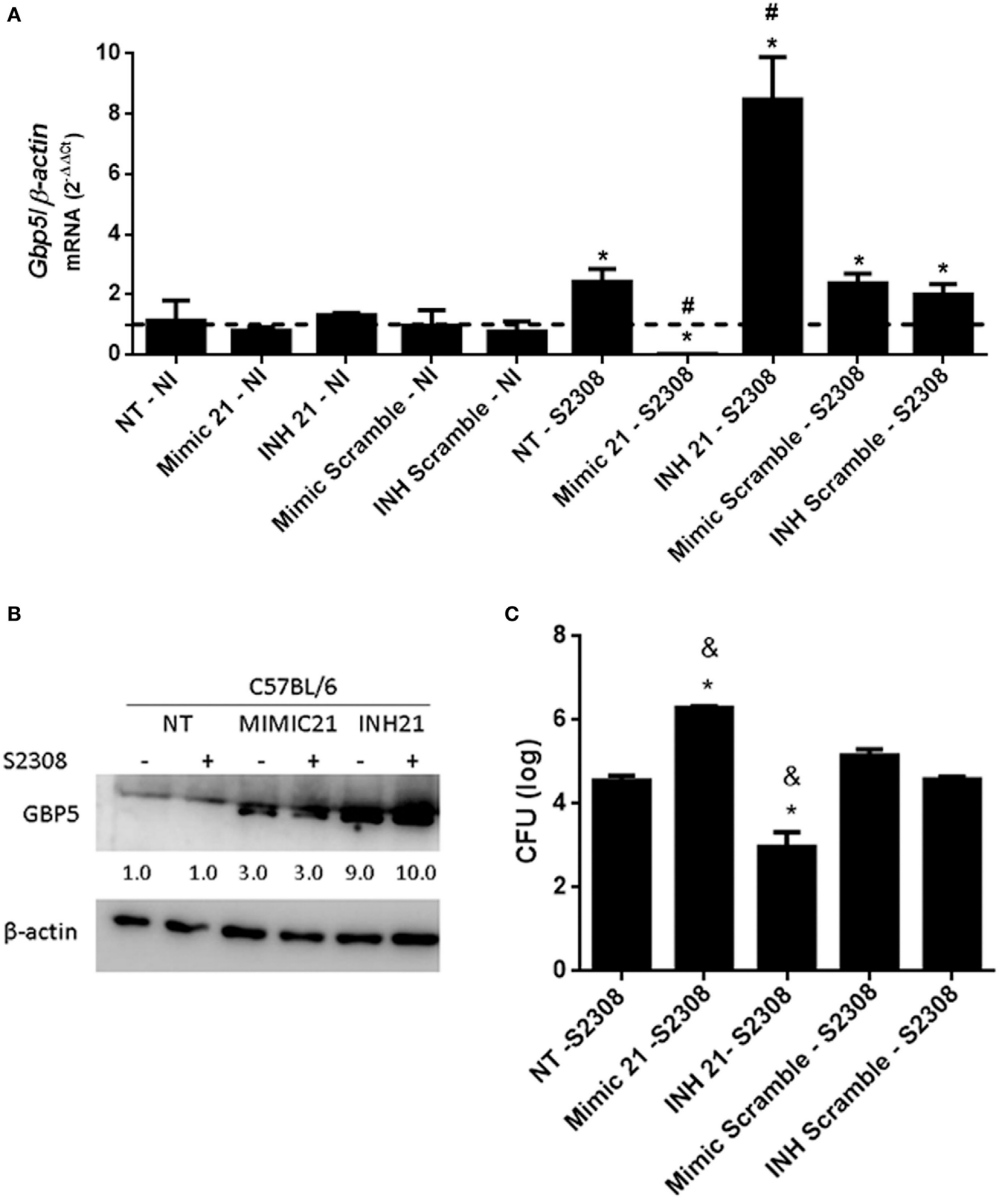
mmu-miR-21a-5p modulates GPB5 expression and *Brucella abortus* infection in macrophages. Bone marrow-derived macrophages (BMDMs) transfected with mimic or inhibitor for mmu-miR-21a-5p were infected with *B. abortus* and the level of guanylate-binding protein (GBP)5 mRNA was measured by real-time PCR **(A)**. The results were normalized to *β-actin*. Error bars represent the mean ± SD. Similar results were obtained in two independent experiments. Statistically significant differences of *GBP5* expression from infected cells to non-transfected (NT)/non-infected (NI) cells are denoted by an asterisk (*p* < 0.05), and # (*p* < 0.05) represents statistically significant differences compared with NT/infected (S2308) cells. **(B)** Expression of GBP5 protein in cytosol from NI and *B. abortus*-infected BMDMs was assessed by immunoblotting assay. BMDMs were transfected with mimic (MIMIC21) or inhibitors (INH21) for mmu-miR-21a-5p in C57BL/6 cells. The symbol + indicates cells infected with *B. abortus* S2308 and − indicates NI cells. β-actin was used as a loading control. Values below each band indicate the quantification of band intensity relative to the loading control. Data are representative of two independent experiments. **(C)** Intracellular growth of *B. abortus* in macrophages transfected with mimics or inhibitor was assessed by CFU analysis at 24 h post-infection. Error bars represent the mean ± SD. Statistically significant differences compared with NT/S2308 cells are denoted by an asterisk (*p* < 0.05), and & (*p* < 0.05) represents statistically significant differences compared with scramble controls.

In addition to these previously characterized targets, we also observed that the mRNA for several GBPs including GBP2, 4, 5, and 8 had binding sites for mmu-miR-21a-5p (Table S3 in Supplementary Material). GBPs are interferon-inducible GTPases that exert direct anti-microbial effects (31). GBPs encoded by genes on mouse chromosome 3 (GBP1, GBP2, GBP3, GBP5, and GBP7) promote recognition of the vacuolar bacterium *Salmonella typhimurium* leading to the escape of the bacteria into the cytosol (32). More recently, our group showed that the same GBPs are critical for *B. abortus* control in mouse macrophages and *in vivo* (33). Since GBPs are putative targets of miR-21a-5p, we decided to evaluate whether this miRNA could also regulate *GBP5* expression in macrophages infected with *B. abortus*. Transfection of the miR-21a-5p mimic decreased levels of the *GBP5* mRNA in macrophages (Figure 7A). By contrast, cells transfected with miR-21a-5p inhibitor showed increased levels of *GBP5* transcripts (Figure 7A). We also assessed GBP5 protein levels in BMDMs treated with miR-21a-5p mimic or inhibitor. As shown in Figure 7B, it was possible to observe that *B. abortus*-infected cells transfected with the inhibitor for mmu-miR-21a-5p showed an enhanced amount of GBP5 when compared with untreated cells. Surprisingly, we also detected an increased in GBP5 in cells treated with the mimic for mmu-miR-21a-5p but in much lower levels compared with macrophages transfected with the inhibitor. Since GBPs are important mediators of anti-bacterial immunity, we decided to investigate whether miR-21a-5p affects *Brucella* replication in macrophages. To evaluate the influence of miR-21a-5p in intracellular *B. abortus* growth in macrophages, BMDMs were transfected with mimic or inhibitor and infected with *B. abortus* for 24 hrs. CFU analysis demonstrated that the miR-21a-5p mimic increased numbers of intracellular bacteria while the inhibitor had the opposite effect (Figure 7C). Taken together, these results suggest that miR-21a-5p modulates *GBP5* expression, thus affecting the ability of the host to control *B. abortus* infection.

## Discussion

MicroRNAs play important role in the regulation of immune response to different infectious agents (10). *B. abortus* is a facultative intracellular bacterium that can adapt to environmental stressor and replicates in phagocytic cells. Many strategies are used by bacteria to evade immune response and survive inside phagocytes, and recently it was characterized novel virulence-related sRNA in *B. melitensis* (34). However, the host triggers intracellular signaling pathways to induce an effective immune response (35). In this battle between host and pathogen, many regulators are induced, including miRNAs. During *B. melitensis* infection in Raw264.7 cells, 57 miRNAs were differentially expressed and they potentially play important regulatory roles in the *Brucella*–host interactions (24). Among these 57, 3 miRNAs (miR-145a-3p, miR-146b-5p, and miR-151a-3p) were also identified as differentially expressed in our study. However, they showed a different profile. miR-145a-3p and miR-146b-5p were upregulated in *B. melitensis*-infected Raw264.7 cells and downregulated in *B. abortus*-infected BMDMs. In addition, miR-151a-3p was downregulated in *B. melitensis*-infected Raw264.7 cells and upregulated in *B. abortus*-infected BMDMs. These differences could be related to the virulence of the *Brucella* species studied or the source of host cells used. Macrophages sense *B. abortus* and induce upregulation of *IL-12, IL-1β, TNF-α*, and *IL-6* genes as early as 30 min after infection. This acute inflammatory response against *B. abortus* has been receiving attention by our group since this bacterium is rapidly recognized by several innate immunity receptors such as TLRs (25) and inflammasomes (36). Therefore, we decided to identify miRNAs that could be related to the initial phase of immune response against *B. abortus* using high-throughput sequencing of small RNAs. RNAseq indicated that 69 miRNAs were differentially expressed between infected and control macrophages, 38 upregulated and 31 downregulated. According to the expression levels and fold-change comparing *Brucella*-infected versus NI libraries, we selected four miRNAs that were upregulated (mmu-miR-151-3p, mmu-miR-155-5p, mmu-miR-181a-5p, and mmu-miR-328-3p) and five miRNAs that were downregulated (mmu-miR-21a-5p, mmu-miR-98-5p, mmu-miR-145a-5p, mmu-miR-146b-5p, and mmu-miR-374b-5p) for validation and further analysis. By qPCR, we validated the differential expression of four upregulated and five downregulated miRNAs in *Brucella*-infected macrophages or spleen cells thus confirming the profile observed in the RNAseq analysis.

Previously, we (25) and others (37) have shown the critical role of MyD88 adaptor molecule in triggering innate immune responses against *B. abortus* and *B. melitensis*, respectively. Since MyD88 pathway is important for control of *Brucella* infection, we evaluated the role of this adaptor molecule in the differential expression of miRNAs. We observed a dependence of MyD88 in upregulation of mmu-miR-181a-5p, while it was not observed differences of upregulation of mmu-miR-328-3p in the absence of MyD88. On the other hand, we observed a dependence of MyD88 for the downregulation of mmu-miR-21a-5p, mmu-miR-98-5p, and mmu-miR-146b-5p during *B. abortus* infection in macrophages. Furthermore, analysis of MyD88 regulation of miRNAs expression *ex vivo* in mouse spleens revealed that among upregulated miRNAs, only mmu-miR-181a-5p shows the same profile in macrophages and *ex vivo*. However, among all downregulated miRNAs tested, the profile observed was similar *in vitro* and *ex vivo* highlighting the importance of MyD88 in regulating miRNA expression. Even though we did not study the involvement of individual TLRs in regulating the miRNAs tested here, we hypothesize that TLR9 may play a role since we have previously demonstrated this is the most critical TLR involved in *B. abortus* infection (38). Recently, Jentho et al. (39) characterized miRNA regulation during *Legionella pneumophila* infection and demonstrated that miR-125a-3p expression is downregulated in a MyD88-dependent manner thus affecting *Legionella*–host cell interactions. These results demonstrate the importance of MyD88 in regulating miRNA expression and the host response during bacterial infections.

We carried out further functional analysis using mmu-miR-181a-5p and mmu-miR-21a-5p. Recently, Luo et al. (29) have identified that *B. suis* upregulates miR-146a, miR-181a, miR-181b, and miR-301a-3p leading to reduced TNF-α expression in Raw264.7 cells. In addition, according to Dan et al. (40), stability of *TNF-α* mRNA was influenced by miR-181a-5p. Therefore, we tested the effect of miR-181a-5p mimic or inhibitor in *TNF-α* expression during *Brucella-*infected BMDMs. miR-181a-5p mimic diminished *TNF-α* expression whereas miRNA inhibitor increased *TNF-α* transcripts. These results corroborate the ability of miR-181a-5p to downregulate TNF-α in *Brucella*-infected BMDMs. As for mmu-miR-21a-5p, one defined target is PDCD4, which is a suppressor of the anti-inflammatory cytokine IL-10. Cohen and Prince (41) showed that during bacterial pneumonia type III IFNλ promotes inflammation by inhibiting miR-21, upregulating PDCD4, and consequently diminishing IL-10 production. PDCD4 influences IL-10 mRNA stability leading to a reduced production of this anti-inflammatory cytokine (42). Therefore, we decided to test the effect of miR-21a-5p mimic or inhibitor in *IL-10* expression on *Brucella*-infected macrophages. Herein, we show that when miR-21a-5p mimic was used higher levels of *IL-10* mRNA were detected in infected cells compared with the controls. By contrast, when the cells were transfected with miR-21a-5p inhibitor, we observed a strong reduction in *IL-10* levels. This result suggests the role of miR-21a-5p in controlling *IL-10* expression *via* PDCD4 during *B. abortus* infection. Previously, we and others demonstrated that IL-10 modulates the pro-inflammatory immune response to *B. abortus* and the lack of IL-10 increases resistance to *Brucella* infection (8, 9).

Recently, we have determined that GBPs encoded by genes on mouse chromosome 3 (GBP1, GBP2, GBP3, GBP5, and GBP7) are critical for *B. abortus* control in macrophages and in mice (33). Therefore, we decided to search for GBP genes as potential targets for miR-181a-5p or miR-21a-5p regulation. We found that GBP2, GBP4, GBP5, and GBP8 are putative targets for miR-21a-5p but not for miR-181a-5p. We then selected GBP5 as a target to evaluate the potential regulatory role of miR-21a-5p. When miR-21a-5p mimic was transfected in macrophages, we observed reduced *GBP5* expression. By contrast, the miR-21a-5p inhibitor increased *GBP5* transcripts. As for GBP5 protein, miR-21a-5p inhibitor enhanced GBP5 levels in infected and uninfected macrophages compared with miR-21a-5p mimic treated cells. Unexpectedly, we observed a small increase in GBP5 in the Western blot when cells were transfected with miR-21a-5p mimic. It is possible that the transfection reagent induced a background level of GBP5 that was detected by the polyclonal antibody. These results show that *GBP5* expression is regulated by miR-21a-5p. In addition, pretreatment of BMDMs with miR-21a-5p inhibitor significantly decreased the intracellular *Brucella* numbers upon bacterial infection. By contrast, miR-21a-5p mimic pretreatment increased bacterial load in macrophages.

**Figure 8:**
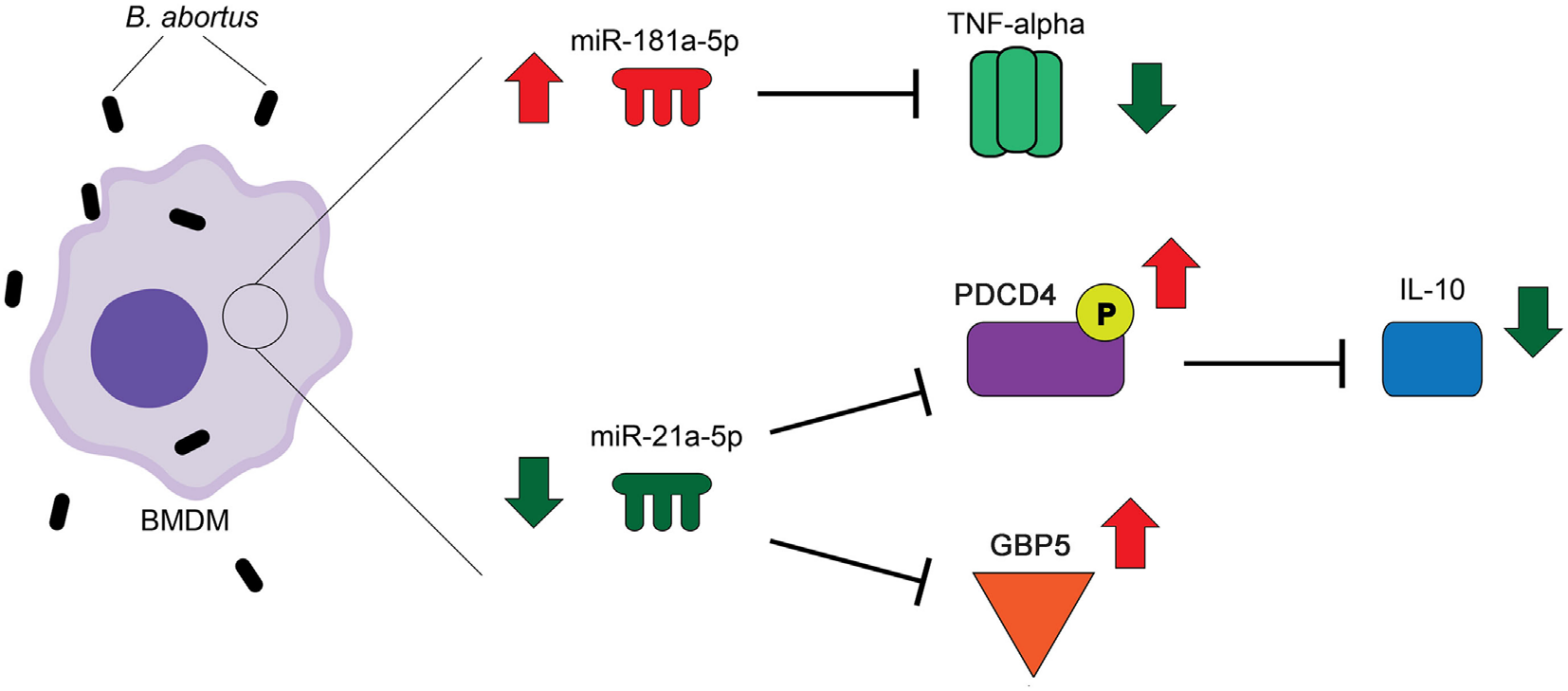
Schematic model of the role of miR-181a-5p and miR-21a-5p during *Brucella abortus* infection. The intracellular bacteria *B. abortus* enters the host cell and upregulates miR-181a-5p and downregulates miR-21-5p. miR-181a-5p regulates *TNF-α* and miR-21a-5p influences *IL-10* expression during bacterial infection. In addition, miR-21a-5p also regulates guanylate-binding protein (*GBP*)*5* transcription and protein production in macrophages thus affecting *Brucella* intracellular growth.

In summary, the findings present here provide evidences that miR-181a-5p regulates *TNF-α* and miR-21a-5p influences *IL-10* expression during *B. abortus* infection (Figure 8). In addition, miR-21a-5p also regulates *GBP5* transcription and protein production in macrophages thus affecting *Brucella* intracellular growth. Herein, we hypothesize that in initial phase of infection host cells can downregulate miR-21a-5p expression to reduce *IL-10* and increase *GBP5* expression thus resulting in improved control of *Brucella* replication.

## Ethics Statement

This study was carried out in strict accordance with the Brazilian laws 6638 and 9605 in Animal Experimentation. The protocol was approved by the Committee on the Ethics of Animal Experiments of the Federal University of Minas Gerais (Permit Number: CETEA no. 128/2014).

## Author Contributions

PC, LA, JM, and SO designed the project and experiments. PC, LA, MG, EG, and AG carried out most of the experiments. PC, JM, and SO wrote the manuscript. PC, LA, and AG carried out statistical analysis and prepared figures. SO submitted this paper. All the authors reviewed the manuscript.

## Conflict of Interest Statement

The authors declare that the research was conducted in the absence of any commercial or financial relationships that could be construed as a potential conflict of interest.

## Footnotes

^1^https://github.com/marcelm/cutadapt (Accessed: May 14, 2015).

^2^https://www.bioinformatics.babraham.ac.uk/projects/fastqc/ (Accessed: March 25, 2015).

^3^http://bowtie-bio.sourceforge.net/index.shtml (Accessed: June 21, 2015).

^4^http://bedtools.readthedocs.io/en/latest/ (Accessed: June 25, 2015).

^5^http://gtrnadb.ucsc.edu/Mmusc10/mm10-tRNAs.fa, http://gtrnadb.ucsc.edu/genomes/eukaryota/Mmusc10/mm10-tRNAs.tar.gz (Accessed: November 16, 2017).

^6^http://mirwalk.umm.uni-heidelberg.de/ (Accessed: May 9, 2018).

^7^https://reactome.org/ (Accessed: September 13, 2015).
