# Supplementary material for: miR-181a-5p Regulates TNF-α and miR-21a-5p Influences Gualynate-Binding Protein 5 and IL-10 Expression in Macrophages Affecting Host Control of Brucella abortus Infection
Source: Front Immunol. 2018 Jun 11;9:1331. doi: 10.3389/fimmu.2018.01331 (PMC6004377; doi:10.3389/fimmu.2018.01331)
Supplement: Supplementary file 1 [file Table_1.PDF]

Supplementary Table 1. List of primers and their DNA sequences used in this study.

Primer	Forward	Reverse
**IL-12**	5’-TGGTGTCTCCACTCATGG-3’	5’-AGCAGCAGATGTGAGTGG-3’
**IL-1β**	5’-TGACCTGGGCTGTCCAGATG-3’	5’-CTGTCCATTGAGGTGGAGAG-3’
**TNF-α**	5’-CATCTTCTCAAAATTCGAGTGACAA-3’	5’-TGGGAGTAGACAAGGTACAACCC-3’
**IL-6**	5’-CCAGGTAGCTATGGTACTCCAGAA-3’	5’-GATGGATGCTACCAAACTGGA-3’
**IL-10**	5’-GGTTGCCAAGCCTTATCGGA-3’	5’-ACCTGCTCCACTGCCTTGCT-3’
**GBP5**	5’-CTGAACTCAGATTTTGTGCAGGA-3’	5’-CATCGACATAAGTCAGCACCAG-3’
**β-actin**	5’-AGGTGTGCACCTTTTATTGGTCTCAA-3’	5’-TGTATGAAGGTTTGGTCTCCCT-3’
