# Supplementary material for: miR-181a-5p Regulates TNF-α and miR-21a-5p Influences Gualynate-Binding Protein 5 and IL-10 Expression in Macrophages Affecting Host Control of Brucella abortus Infection
Source: Front Immunol. 2018 Jun 11;9:1331. doi: 10.3389/fimmu.2018.01331 (PMC6004377; doi:10.3389/fimmu.2018.01331)
Supplement: Supplementary file 2 [file Table_2.PDF]

Supplementary Table 2. Comparative analysis of miRNA expression in control and *Brucella* infected macrophages.

Feature	Infected (number of reads)	Infected (RPM)	Non-infected (number of reads)	Non-infected (RPM)	Fold Change (Infected/Non-infected)	Regulated
mmu-miR-144-3p	1	0.000039	254	0.0066	0.0059	No change
mmu-miR-451a	8	0.00031	1,732	0.045	0.0069	No change
mmu-miR-144-5p	4	0.00016	557	0.014	0.0107	No change
mmu-miR-136-3p	1	0.000039	24	0.00062	0.0623	No change
mmu-miR-344-3p	1	0.000039	17	0.00044	0.0879	No change
mmu-miR-7018-3p	1	0.000039	14	0.00036	0.1068	No change
mmu-miR-126b-5p	2	0.000078	28	0.00073	0.1068	No change
mmu-miR-19a-5p	1	0.000039	13	0.00034	0.1150	No change
mmu-miR-196a-2-3p	5	0.00019	59	0.0015	0.1267	No change
mmu-miR-6989-3p	1	0.000039	11	0.00029	0.1359	No change
mmu-miR-1298-5p	1	0.000039	11	0.00029	0.1359	No change
mmu-miR-107-5p	1	0.000039	11	0.00029	0.1359	No change
mmu-miR-200b-3p	1	0.000039	11	0.00029	0.1359	No change
mmu-miR-6935-5p	1	0.000039	10	0.00026	0.1495	No change
mmu-miR-6994-3p	1	0.000039	10	0.00026	0.1495	No change
mmu-miR-142b	2	0.000078	20	0.00052	0.1495	No change
mmu-miR-3109-5p	1	0.000039	9	0.00023	0.1661	No change
mmu-miR-3101-5p	2	0.000078	17	0.00044	0.1759	No change
mmu-miR-16-2-3p	16	0.00062	131	0.0034	0.1826	No change
mmu-miR-337-3p	1	0.000039	8	0.000208	0.1868	No change
mmu-miR-6995-5p	1	0.000039	8	0.000208	0.1868	No change
mmu-miR-3084-5p	3	0.00012	23	0.000598	0.1950	No change
mmu-miR-3107-3p	145	0.0056	1,074	0.028	0.2018	No change
mmu-miR-3473a	1	0.000039	7	0.00018	0.2135	No change
mmu-miR-669b-3p	1	0.000039	7	0.00018	0.2135	No change
mmu-miR-7652-3p	2	0.000078	14	0.00036	0.2135	No change
mmu-miR-380-3p	6	0.00023	40	0.0010	0.2242	No change
mmu-miR-7224-3p	2	0.000078	13	0.00034	0.2300	No change
mmu-miR-379-5p	1	0.000039	6	0.00016	0.2491	No change
mmu-miR-7a-5p	1	0.000039	6	0.00016	0.2491	No change
mmu-miR-7670-3p	3	0.00012	18	0.00047	0.2491	No change
mmu-miR-486-5p	7,461	0.29	43,037	1.119	0.2591	Down
mmu-miR-3107-5p	7,461	0.29	43,037	1.119	0.2591	Down
mmu-miR-6998-5p	2	0.000078	11	0.00029	0.2718	No change
mmu-miR-1955-3p	3	0.000117	16	0.00042	0.2803	No change
mmu-miR-5123	3	0.000117	16	0.00042	0.2803	No change
mmu-miR-5615-5p	4	0.000155	21	0.00055	0.2847	No change
mmu-miR-3103-3p	3	0.000117	15	0.00039	0.2989	No change
mmu-miR-505-3p	3	0.000117	15	0.00039	0.2989	No change
mmu-miR-493-5p	3	0.000117	15	0.00039	0.2989	No change
mmu-miR-96-3p	1	0.000039	5	0.00013	0.2989	No change
mmu-miR-323-3p	1	0.000039	5	0.00013	0.2989	No change
mmu-miR-7646-5p	1	0.000039	5	0.00013	0.2989	No change
mmu-miR-16-1-3p	16	0.0006	79	0.0021	0.3027	No change
mmu-miR-1198-3p	6	0.000233	28	0.000728	0.3203	No change
mmu-miR-301a-5p	25	0.0010	116	0.0030	0.3221	No change
mmu-miR-335-3p	70	0.0027	317	0.0082	0.3301	No change
mmu-miR-215-5p	2	0.000078	9	0.000234	0.3322	No change
mmu-miR-7664-3p	2	0.000078	9	0.000234	0.3322	No change
mmu-miR-3963	4	0.000155	18	0.000468	0.3322	No change
mmu-miR-219c-5p	8	0.0003	34	0.000884	0.3517	No change
mmu-miR-7093-5p	6	0.000233	25	0.000650	0.3587	No change
mmu-miR-363-3p	10	0.0004	41	0.0011	0.3646	No change
mmu-miR-3069-3p	3	0.000117	12	0.000312	0.3737	No change
mmu-miR-1249-5p	1	0.000039	4	0.000104	0.3737	No change
mmu-miR-701-3p	1	0.000039	4	0.000104	0.3737	No change
mmu-miR-374b-5p	216	0.0084	823	0.0214	0.3923	Down

mmu-miR-6928-5p	3	0.000117	11	0.000286	0.4077	No change
mmu-miR-3066-5p	3	0.000117	11	0.000286	0.4077	No change
mmu-miR-200a-3p	15	0.0006	53	0.0014	0.4230	No change
mmu-miR-6913-3p	4	0.000155	14	0.000364	0.4271	No change
mmu-miR-15b-3p	96	0.0037	335	0.0087	0.4283	No change
mmu-miR-183-3p	9	0.000350	31	0.000806	0.4340	No change
mmu-miR-145a-3p	527	0.0205	1,797	0.0467	0.4384	Down
mmu-miR-369-3p	8	0.000311	27	0.000702	0.4429	No change
mmu-miR-26a-2-3p	62	0.0024	204	0.0053	0.4543	No change
mmu-miR-154-5p	4	0.000155	13	0.000338	0.4599	No change
mmu-miR-1964-5p	4	0.000155	13	0.000338	0.4599	No change
mmu-miR-3081-3p	4	0.000155	13	0.000338	0.4599	No change
mmu-miR-1933-5p	6	0.000233	19	0.000494	0.4720	No change
mmu-miR-7091-3p	12	0.0005	38	0.0010	0.4720	No change
mmu-miR-126a-5p	12,421	0.4825	39,318	1.0218	0.4722	Down
mmu-miR-126b-3p	12,430	0.4829	39,334	1.0223	0.4724	Down
mmu-miR-3065-5p	57	0.0022	180	0.0047	0.4733	No change
mmu-miR-218-5p	93	0.0036	292	0.0076	0.4761	No change
mmu-miR-190a-5p	8	0.000311	25	0.000650	0.4783	No change
mmu-miR-142-5p	11,712	0.4550	36,265	0.9425	0.4827	Down
mmu-miR-450a-5p	276	0.0107	854	0.0222	0.4831	Down
mmu-miR-411-5p	708	0.0275	2,166	0.0563	0.4886	Down
mmu-miR-6899-5p	1	0.000039	3	0.000078	0.4982	No change
mmu-miR-6912-5p	1	0.000039	3	0.000078	0.4982	No change
mmu-miR-6977-5p	1	0.000039	3	0.000078	0.4982	No change
mmu-miR-8097	1	0.000039	3	0.000078	0.4982	No change
mmu-miR-667-5p	1	0.000039	3	0.000078	0.4982	No change
mmu-miR-693-3p	1	0.000039	3	0.000078	0.4982	No change
mmu-miR-329-5p	2	0.000078	6	0.000156	0.4982	No change
mmu-miR-1929-5p	2	0.000078	6	0.000156	0.4982	No change
mmu-miR-297b-5p	4	0.000155	12	0.000312	0.4982	No change
mmu-miR-3102-5p	4	0.000155	12	0.000312	0.4982	No change
mmu-miR-1966-5p	8	0.000311	24	0.000624	0.4982	No change
mmu-miR-1b-3p	3	0.000117	9	0.000234	0.4982	No change
mmu-miR-133a-3p	10	0.000388	30	0.000780	0.4982	No change
mmu-miR-93-3p	10	0.000388	30	0.000780	0.4982	No change
mmu-miR-6960-5p	20	0.0008	60	0.0016	0.4982	No change
mmu-miR-301a-3p	11,884	0.4617	35,511	0.9229	0.5002	Down
mmu-miR-20a-5p	480	0.0186	1,408	0.0366	0.5096	Down
mmu-miR-19b-3p	3,895	0.1513	11,211	0.2914	0.5193	Down
mmu-miR-141-3p	123	0.0048	353	0.0092	0.5208	No change
mmu-miR-126a-3p	2,787	0.1083	7,950	0.2066	0.5240	Down
mmu-miR-18a-5p	157	0.0061	447	0.0116	0.5250	No change
mmu-miR-146b-5p	1,022	0.0397	2,831	0.0736	0.5396	Down
mmu-miR-101a-5p	121	0.0047	333	0.0087	0.5431	No change
mmu-miR-150-3p	4	0.000155	11	0.000286	0.5435	No change
mmu-miR-709	19	0.0007	52	0.0014	0.5462	No change
mmu-miR-6537-3p	38	0.0015	103	0.0027	0.5515	No change
mmu-miR-299a-5p	3	0.000117	8	0.000208	0.5605	No change
mmu-miR-128-3p	2,181	0.0847	5,799	0.1507	0.5622	Down
mmu-miR-21a-5p	756,779	29.3988	1,998,189	51.9317	0.5661	Down
mmu-miR-344b-3p	8	0.000311	21	0.000546	0.5694	No change
mmu-miR-1943-3p	5	0.000194	13	0.000338	0.5749	No change
mmu-miR-669d-5p	10	0.000388	26	0.000676	0.5749	No change
mmu-miR-582-3p	40	0.0016	103	0.0027	0.5805	No change
mmu-miR-7219-3p	9	0.000350	23	0.000598	0.5849	No change
mmu-miR-486-3p	472	0.0183	1,193	0.0310	0.5914	Down
mmu-miR-8103	25	0.0010	63	0.0016	0.5932	No change
mmu-miR-15a-5p	10,618	0.4125	26,741	0.6950	0.5935	Down
mmu-miR-1247-3p	463	0.0180	1,160	0.0301	0.5966	Down
mmu-miR-30a-5p	6,599	0.2564	16,510	0.4291	0.5974	Down
mmu-let-7f-1-3p	10	0.000388	25	0.000650	0.5979	No change
mmu-miR-7213-5p	12	0.000466	30	0.000780	0.5979	No change
mmu-miR-3079-3p	2	0.000078	5	0.000130	0.5979	No change

mmu-miR-7649-3p	4	0.000155	10	0.000260	0.5979	No change
mmu-miR-129-2-3p	164	0.0064	410	0.0107	0.5979	No change
mmu-miR-34b-5p	288	0.0112	718	0.0187	0.5996	Down
mmu-miR-335-5p	94	0.0037	234	0.0061	0.6005	No change
mmu-miR-98-5p	19,958	0.7753	48,785	1.2679	0.6115	Down
mmu-miR-132-5p	32	0.0012	78	0.0020	0.6132	No change
mmu-miR-362-3p	168	0.0065	407	0.0106	0.6170	No change
mmu-let-7e-3p	121	0.0047	292	0.0076	0.6194	No change
mmu-miR-467a-5p	170	0.0066	409	0.0106	0.6213	No change
mmu-miR-467e-5p	56	0.0022	134	0.0035	0.6247	No change
mmu-miR-29b-3p	341	0.0132	811	0.0211	0.6285	Down
mmu-miR-150-5p	185	0.0072	439	0.0114	0.6299	No change
mmu-miR-429-3p	44	0.0017	103	0.0027	0.6385	No change
mmu-miR-6936-3p	3	0.000117	7	0.000182	0.6406	No change
mmu-miR-7682-3p	3	0.000117	7	0.000182	0.6406	No change
mmu-miR-16-5p	341,873	13.2809	795,859	20.6839	0.6421	Down
mmu-miR-450b-5p	110	0.0043	256	0.0067	0.6423	No change
mmu-miR-3068-3p	848	0.0329	1,968	0.0511	0.6441	Down
mmu-miR-10b-5p	15,875	0.6167	36,705	0.9539	0.6465	Down
mmu-miR-19a-3p	399	0.0155	919	0.0239	0.6490	Down
mmu-miR-467d-5p	225	0.0087	517	0.0134	0.6505	Down
mmu-miR-148a-5p	2,893	0.1124	6,619	0.1720	0.6533	Down
mmu-miR-421-3p	2,167	0.0842	4,922	0.1279	0.6581	Down
mmu-miR-22-3p	3,117,121	121.0918	7,070,647	183.7616	0.6590	Down
mmu-let-7f-2-3p	4	0.000155	9	0.000234	0.6643	No change
mmu-miR-452-5p	8	0.000311	18	0.000468	0.6643	No change
mmu-miR-187-3p	22	0.0009	49	0.0013	0.6711	No change
mmu-miR-467b-5p	66	0.0026	147	0.0038	0.6711	No change
mmu-miR-30d-3p	1,525	0.0592	3,391	0.0881	0.6722	No change
mmu-miR-872-5p	4,066	0.1580	9,024	0.2345	0.6735	No change
mmu-miR-338-3p	1,753	0.0681	3,880	0.1008	0.6753	No change
mmu-miR-32-3p	24	0.0009	53	0.0014	0.6769	No change
mmu-miR-379-3p	5	0.000194	11	0.000286	0.6794	No change
mmu-miR-5122	10	0.000388	22	0.000572	0.6794	No change
mmu-miR-434-5p	107	0.0042	235	0.0061	0.6806	No change
mmu-miR-143-3p	790,613	30.7132	1,735,737	45.1107	0.6808	No change
mmu-miR-222-5p	601	0.0233	1,317	0.0342	0.6821	No change
mmu-miR-7235-3p	37	0.0014	81	0.0021	0.6828	No change
mmu-miR-199b-3p	58,245	2.2627	127,241	3.3069	0.6842	No change
mmu-miR-199a-3p	58,245	2.2627	127,241	3.3069	0.6842	No change
mmu-miR-106b-5p	1,318	0.0512	2,870	0.0746	0.6864	No change
mmu-let-7f-5p	812,176	31.5509	1,764,741	45.8645	0.6879	No change
mmu-miR-17-5p	1,898	0.0737	4,079	0.1060	0.6955	No change
mmu-miR-7668-5p	7	0.000272	15	0.000390	0.6975	No change
mmu-miR-299a-3p	7	0.000272	15	0.000390	0.6975	No change
mmu-miR-1199-3p	7	0.000272	15	0.000390	0.6975	No change
mmu-miR-3068-5p	51	0.0020	109	0.0028	0.6994	No change
mmu-miR-223-3p	13,580	0.5275	28,960	0.7527	0.7009	No change
mmu-miR-449a-5p	24	0.0009	51	0.0013	0.7034	No change
mmu-miR-130a-3p	6,055	0.2352	12,836	0.3336	0.7051	No change
mmu-miR-301b-3p	1,005	0.0390	2,127	0.0553	0.7063	No change
mmu-miR-199b-5p	43	0.0017	91	0.0024	0.7063	No change
mmu-miR-455-5p	165	0.0064	348	0.0090	0.7087	No change
mmu-miR-344d-3p	132	0.0051	278	0.0072	0.7097	No change
mmu-miR-1969	13	0.000505	27	0.000702	0.7197	No change
mmu-miR-1956	29	0.0011	60	0.0016	0.7225	No change
mmu-miR-93-5p	24,336	0.9454	50,052	1.3008	0.7268	No change
mmu-miR-26b-5p	9,043	0.3513	18,401	0.4782	0.7346	No change
mmu-miR-7a-2-3p	3	0.000117	6	0.000156	0.7474	No change
mmu-miR-7663-3p	3	0.000117	6	0.000156	0.7474	No change
mmu-miR-7236-3p	3	0.000117	6	0.000156	0.7474	No change
mmu-miR-378d	3	0.000117	6	0.000156	0.7474	No change
mmu-miR-19b-1-5p	3	0.000117	6	0.000156	0.7474	No change
mmu-miR-7683-5p	7	0.000272	14	0.000364	0.7474	No change

mmu-miR-7024-5p	1	0.000039	2	0.000052	0.7474	No change
mmu-miR-592-5p	1	0.000039	2	0.000052	0.7474	No change
mmu-miR-669k-5p	1	0.000039	2	0.000052	0.7474	No change
mmu-miR-7064-5p	1	0.000039	2	0.000052	0.7474	No change
mmu-miR-5616-3p	1	0.000039	2	0.000052	0.7474	No change
mmu-miR-6399	1	0.000039	2	0.000052	0.7474	No change
mmu-miR-7658-3p	2	0.000078	4	0.000104	0.7474	No change
mmu-miR-181d-3p	2	0.000078	4	0.000104	0.7474	No change
mmu-miR-7653-5p	2	0.000078	4	0.000104	0.7474	No change
mmu-miR-18b-5p	2	0.000078	4	0.000104	0.7474	No change
mmu-miR-7019-3p	2	0.000078	4	0.000104	0.7474	No change
mmu-miR-6937-3p	2	0.000078	4	0.000104	0.7474	No change
mmu-miR-467c-3p	2	0.000078	4	0.000104	0.7474	No change
mmu-miR-31-3p	4	0.000155	8	0.000208	0.7474	No change
mmu-miR-466i-5p	4	0.000155	8	0.000208	0.7474	No change
mmu-miR-1950	5	0.000194	10	0.000260	0.7474	No change
mmu-miR-7651-5p	8	0.000311	16	0.000416	0.7474	No change
mmu-miR-1938	9	0.000350	18	0.000468	0.7474	No change
mmu-miR-6983-3p	9	0.000350	18	0.000468	0.7474	No change
mmu-miR-7033-5p	16	0.0006	32	0.000832	0.7474	No change
mmu-miR-32-5p	83	0.0032	166	0.0043	0.7474	No change
mmu-miR-3535	128	0.0050	255	0.0066	0.7503	No change
mmu-miR-501-5p	47	0.0018	93	0.0024	0.7554	No change
mmu-miR-872-3p	2,215	0.0860	4,352	0.1131	0.7608	No change
mmu-miR-3074-2-3p	70	0.0027	137	0.0036	0.7637	No change
mmu-miR-194-5p	1,084	0.0421	2,119	0.0551	0.7647	No change
mmu-miR-148b-5p	1,253	0.0487	2,442	0.0635	0.7670	No change
mmu-miR-186-3p	19	0.0007	37	0.0010	0.7676	No change
mmu-miR-193a-3p	289	0.0112	562	0.0146	0.7686	No change
mmu-miR-223-5p	398	0.0155	773	0.0201	0.7696	No change
mmu-miR-10a-5p	424,712	16.4989	823,014	21.3896	0.7714	No change
mmu-miR-3061-3p	73	0.0028	140	0.0036	0.7794	No change
mmu-miR-411-3p	12	0.000466	23	0.000598	0.7799	No change
mmu-miR-96-5p	24	0.0009	46	0.0012	0.7799	No change
mmu-miR-3110-5p	23	0.0009	44	0.0011	0.7813	No change
mmu-miR-30e-5p	1,744	0.0677	3,334	0.0866	0.7819	No change
mmu-miR-22-5p	504	0.0196	963	0.0250	0.7823	No change
mmu-let-7i-5p	572,113	22.2251	1,092,450	28.3921	0.7828	No change
mmu-miR-1949	11	0.000427	21	0.000546	0.7830	No change
mmu-miR-7667-3p	22	0.0009	42	0.0011	0.7830	No change
mmu-miR-29a-5p	98	0.0038	187	0.0049	0.7833	No change
mmu-let-7g-5p	80,820	3.1396	152,177	3.9550	0.7938	No change
mmu-miR-542-3p	185	0.0072	348	0.0090	0.7946	No change
mmu-miR-3105-3p	8	0.000311	15	0.000390	0.7972	No change
mmu-miR-1945	16	0.000622	30	0.000780	0.7972	No change
mmu-miR-101b-3p	554	0.0215	1,033	0.0268	0.8016	No change
mmu-let-7c-2-3p	180	0.0070	335	0.0087	0.8031	No change
mmu-let-7a-1-3p	180	0.0070	335	0.0087	0.8031	No change
mmu-miR-3473d	92	0.0036	171	0.0044	0.8042	No change
mmu-miR-193a-5p	7	0.000272	13	0.000338	0.8049	No change
mmu-miR-301b-5p	7	0.000272	13	0.000338	0.8049	No change
mmu-miR-1191	62	0.0024	115	0.0030	0.8059	No change
mmu-miR-340-5p	40,833	1.5863	75,505	1.9623	0.8084	No change
mmu-miR-433-3p	26	0.0010	48	0.0012	0.8097	No change
mmu-miR-3065-3p	201	0.0078	370	0.0096	0.8120	No change
mmu-miR-148a-3p	73,603	2.8593	135,205	3.5139	0.8137	No change
mmu-miR-33-5p	29	0.0011	53	0.0014	0.8179	No change
mmu-miR-3079-5p	17	0.000660	31	0.000806	0.8197	No change
mmu-miR-152-5p	4,104	0.1594	7,462	0.1939	0.8221	No change
mmu-miR-181d-5p	983	0.0382	1,784	0.0464	0.8236	No change
mmu-miR-212-3p	306	0.0119	555	0.0144	0.8241	No change
mmu-miR-29c-3p	1,964	0.0763	3,559	0.0925	0.8249	No change
mmu-miR-6901-5p	31	0.0012	56	0.0015	0.8274	No change
mmu-miR-712-5p	25	0.0010	45	0.0012	0.8304	No change

mmu-miR-1247-5p	110	0.0043	197	0.0051	0.8346	No change
mmu-miR-350-3p	86	0.0033	154	0.0040	0.8347	No change
mmu-miR-212-5p	171	0.0066	306	0.0080	0.8353	No change
mmu-miR-702-5p	65	0.0025	116	0.0030	0.8376	No change
mmu-miR-466c-5p	18	0.0007	32	0.000832	0.8408	No change
mmu-miR-381-3p	211	0.0082	375	0.0097	0.8410	No change
mmu-miR-24-2-5p	11,293	0.4387	20,054	0.5212	0.8417	No change
mmu-miR-7054-5p	31	0.0012	55	0.0014	0.8425	No change
mmu-miR-1983	387	0.0150	685	0.0178	0.8445	No change
mmu-miR-669f-5p	13	0.000505	23	0.000598	0.8449	No change
mmu-miR-26a-5p	508,731	19.7628	897,166	23.3168	0.8476	No change
mmu-miR-28c	4	0.000155	7	0.000182	0.8541	No change
mmu-miR-26a-1-3p	8	0.000311	14	0.000364	0.8541	No change
mmu-miR-100-3p	8	0.000311	14	0.000364	0.8541	No change
mmu-miR-140-5p	1,335	0.0519	2,333	0.0606	0.8553	No change
mmu-miR-1934-5p	55	0.0021	96	0.0025	0.8564	No change
mmu-miR-34c-5p	40,380	1.5687	70,389	1.8294	0.8575	No change
mmu-miR-6955-5p	19	0.0007	33	0.000858	0.8606	No change
mmu-miR-3058-3p	11	0.000427	19	0.000494	0.8654	No change
mmu-miR-5104	18	0.000699	31	0.000806	0.8679	No change
mmu-miR-101a-3p	4,154	0.1614	7,125	0.1852	0.8715	No change
mmu-miR-24-1-5p	52	0.0020	89	0.0023	0.8733	No change
mmu-miR-138-5p	212	0.0082	358	0.0093	0.8852	No change
mmu-miR-29c-5p	16	0.000622	27	0.000702	0.8858	No change
mmu-miR-9-5p	130	0.0051	217	0.0056	0.8955	No change
mmu-miR-7661-3p	3	0.000117	5	0.000130	0.8968	No change
mmu-miR-409-3p	287	0.0111	474	0.0123	0.9050	No change
mmu-miR-1291	20	0.0008	33	0.000858	0.9059	No change
mmu-miR-188-3p	74	0.0029	122	0.0032	0.9066	No change
mmu-miR-322-5p	9,761	0.3792	16,085	0.4180	0.9071	No change
mmu-miR-147-3p	150	0.0058	247	0.0064	0.9077	No change
mmu-miR-1843b-5p	2,621	0.1018	4,312	0.1121	0.9086	No change
mmu-miR-7a-1-3p	136	0.0053	223	0.0058	0.9116	No change
mmu-miR-1968-5p	27	0.0010	44	0.0011	0.9172	No change
mmu-miR-34b-3p	602	0.0234	981	0.0255	0.9173	No change
mmu-miR-714	170	0.0066	277	0.0072	0.9173	No change
mmu-miR-300-3p	115	0.0045	187	0.0049	0.9192	No change
mmu-miR-222-3p	1,418	0.0551	2,305	0.0599	0.9195	No change
mmu-miR-184-3p	56	0.0022	91	0.0024	0.9198	No change
mmu-miR-219a-1-3p	85	0.0033	138	0.0036	0.9207	No change
mmu-miR-511-3p	2,407	0.0935	3,896	0.1013	0.9235	No change
mmu-let-7c-1-3p	188	0.0073	304	0.0079	0.9244	No change
mmu-miR-192-5p	21,871	0.8496	35,347	0.9186	0.9249	No change
mmu-miR-142-3p	1,893	0.0735	3,059	0.0795	0.9250	No change
mmu-miR-667-3p	5	0.000194	8	0.000208	0.9342	No change
mmu-miR-466n-3p	5	0.000194	8	0.000208	0.9342	No change
mmu-miR-615-3p	2,924	0.1136	4,677	0.1216	0.9345	No change
mmu-miR-129b-5p	32	0.0012	51	0.0013	0.9379	No change
mmu-miR-3082-3p	115	0.0045	183	0.0048	0.9393	No change
mmu-miR-744-3p	371	0.0144	589	0.0153	0.9415	No change
mmu-miR-128-1-5p	349	0.0136	554	0.0144	0.9416	No change
mmu-miR-3473b	7,846	0.3048	12,424	0.3229	0.9440	No change
mmu-miR-434-3p	1,024	0.0398	1,621	0.0421	0.9442	No change
mmu-miR-3473e	7,826	0.3040	12,387	0.3219	0.9444	No change
mmu-miR-10a-3p	397	0.0154	625	0.0162	0.9495	No change
mmu-miR-340-3p	318	0.0124	500	0.0130	0.9507	No change
mmu-miR-6928-3p	7	0.000272	11	0.000286	0.9512	No change
mmu-miR-6988-3p	7	0.000272	11	0.000286	0.9512	No change
mmu-miR-673-3p	14	0.000544	22	0.000572	0.9512	No change
mmu-miR-671-3p	2,139	0.0831	3,357	0.0872	0.9524	No change
mmu-miR-221-3p	70,454	2.7369	110,378	2.8687	0.9541	No change
mmu-miR-450b-3p	30	0.0012	47	0.0012	0.9541	No change
mmu-miR-195a-3p	46	0.0018	72	0.0019	0.9550	No change
mmu-miR-1839-3p	164	0.0064	256	0.0067	0.9576	No change

mmu-miR-8111	25	0.0010	39	0.0010	0.9582	No change
mmu-miR-1839-5p	4,775	0.1855	7,427	0.1930	0.9610	No change
mmu-miR-674-3p	1,596	0.0620	2,479	0.0644	0.9623	No change
mmu-miR-151-5p	13,480	0.5237	20,916	0.5436	0.9633	No change
mmu-miR-181c-5p	6,631	0.2576	10,271	0.2669	0.9650	No change
mmu-let-7j	33	0.0013	51	0.0013	0.9672	No change
mmu-miR-34a-5p	1,837	0.0714	2,838	0.0738	0.9675	No change
mmu-miR-3061-5p	46	0.0018	71	0.0018	0.9684	No change
mmu-miR-541-5p	1,126	0.0437	1,729	0.0449	0.9734	No change
mmu-miR-3109-3p	71	0.0028	109	0.0028	0.9736	No change
mmu-miR-196b-5p	4,197	0.1630	6,397	0.1663	0.9807	No change
mmu-miR-191-3p	550	0.0214	837	0.0218	0.9822	No change
mmu-miR-29a-3p	55,131	2.1417	83,754	2.1767	0.9839	No change
mmu-let-7e-5p	36,067	1.4011	54,594	1.4189	0.9875	No change
mmu-miR-21a-3p	5,356	0.2081	8,107	0.2107	0.9875	No change
mmu-miR-322-3p	957	0.0372	1,447	0.0376	0.9886	No change
mmu-miR-410-3p	671	0.0261	1,007	0.0262	0.9960	No change
mmu-miR-7115-5p	2	0.000078	3	0.000078	0.9965	No change
mmu-miR-6992-5p	2	0.000078	3	0.000078	0.9965	No change
mmu-miR-467e-3p	2	0.000078	3	0.000078	0.9965	No change
mmu-miR-377-5p	2	0.000078	3	0.000078	0.9965	No change
mmu-miR-7029-3p	2	0.000078	3	0.000078	0.9965	No change
mmu-miR-6968-3p	2	0.000078	3	0.000078	0.9965	No change
mmu-miR-6918-5p	2	0.000078	3	0.000078	0.9965	No change
mmu-miR-7036b-5p	4	0.000155	6	0.000156	0.9965	No change
mmu-miR-6911-5p	4	0.000155	6	0.000156	0.9965	No change
mmu-miR-7049-5p	4	0.000155	6	0.000156	0.9965	No change
mmu-miR-23a-5p	8	0.000311	12	0.000312	0.9965	No change
mmu-miR-369-5p	10	0.000388	15	0.000390	0.9965	No change
mmu-miR-1982-3p	12	0.000466	18	0.000468	0.9965	No change
mmu-miR-6933-5p	12	0.000466	18	0.000468	0.9965	No change
mmu-miR-467c-5p	262	0.0102	393	0.0102	0.9965	No change
mmu-miR-145a-5p	5,709	0.2218	8,510	0.2212	1.0028	No change
mmu-miR-1964-3p	1,204	0.0468	1,784	0.0464	1.0088	No change
mmu-miR-181b-5p	87,099	3.3836	128,657	3.3437	1.0119	No change
mmu-miR-30b-5p	25,518	0.9913	37,659	0.9787	1.0128	No change
mmu-miR-1933-3p	347	0.0135	510	0.0133	1.0170	No change
mmu-miR-342-5p	1,403	0.0545	2,062	0.0536	1.0170	No change
mmu-miR-6996-5p	15	0.000583	22	0.000572	1.0191	No change
mmu-miR-25-3p	93,033	3.6141	136,394	3.5448	1.0195	No change
mmu-miR-341-3p	101	0.0039	148	0.0038	1.0201	No change
mmu-miR-431-5p	201	0.0078	294	0.0076	1.0219	No change
mmu-miR-129b-3p	119	0.0046	174	0.0045	1.0223	No change
mmu-miR-26b-3p	1,056	0.0410	1,542	0.0401	1.0236	No change
mmu-miR-7059-5p	208	0.0081	303	0.0079	1.0261	No change
mmu-miR-1948-3p	221	0.0086	321	0.0083	1.0291	No change
mmu-miR-127-3p	5,420	0.2106	7,863	0.2044	1.0303	No change
mmu-miR-362-5p	189	0.0073	274	0.0071	1.0310	No change
mmu-miR-1955-5p	49	0.0019	71	0.0018	1.0316	No change
mmu-miR-146a-5p	73,396	2.8512	106,131	2.7583	1.0337	No change
mmu-miR-3074-5p	16,684	0.6481	24,122	0.6269	1.0338	No change
mmu-miR-877-3p	9	0.000350	13	0.000338	1.0348	No change
mmu-miR-125b-2-3p	5,027	0.1953	7,207	0.1873	1.0426	No change
mmu-miR-1843a-5p	2,759	0.1072	3,951	0.1027	1.0438	No change
mmu-miR-361-5p	1,933	0.0751	2,768	0.0719	1.0438	No change
mmu-miR-6541	7	0.000272	10	0.000260	1.0463	No change
mmu-let-7g-3p	38	0.0015	54	0.0014	1.0519	No change
mmu-miR-6539	298	0.0116	423	0.0110	1.0530	No change
mmu-miR-30d-5p	65,213	2.5334	92,428	2.4021	1.0546	No change
mmu-miR-5100	12	0.000466	17	0.000442	1.0551	No change
mmu-miR-181c-3p	459	0.0178	648	0.0168	1.0588	No change
mmu-miR-3088-3p	15	0.000583	21	0.000546	1.0677	No change
mmu-miR-127-5p	5	0.000194	7	0.000182	1.0677	No change
mmu-miR-6979-3p	5	0.000194	7	0.000182	1.0677	No change

mmu-miR-107-3p	19,626	0.7624	27,410	0.7124	1.0703	No change
mmu-miR-361-3p	2,901	0.1127	4,045	0.1051	1.0720	No change
mmu-miR-210-5p	1,890	0.0734	2,620	0.0681	1.0783	No change
mmu-miR-98-3p	254	0.0099	352	0.0091	1.0786	No change
mmu-miR-92a-3p	9,690	0.3764	13,419	0.3488	1.0794	No change
mmu-miR-186-5p	67,699	2.6299	93,742	2.4363	1.0795	No change
mmu-miR-673-5p	39	0.0015	54	0.0014	1.0795	No change
mmu-miR-3102-3p.2-3p	21	0.000816	29	0.000754	1.0824	No change
mmu-miR-1231-5p	21	0.000816	29	0.000754	1.0824	No change
mmu-miR-669a-5p	455	0.0177	628	0.0163	1.0830	No change
mmu-miR-669p-5p	455	0.0177	628	0.0163	1.0830	No change
mmu-miR-6945-3p	8	0.000311	11	0.000286	1.0871	No change
mmu-miR-7046-3p	8	0.000311	11	0.000286	1.0871	No change
mmu-miR-195a-5p	145	0.0056	199	0.0052	1.0891	No change
mmu-miR-132-3p	2,440	0.0948	3,347	0.0870	1.0897	No change
mmu-let-7d-5p	191,822	7.4518	262,606	6.8250	1.0918	No change
mmu-miR-365-3p	315	0.0122	429	0.0111	1.0975	No change
mmu-miR-23a-3p	22,909	0.8900	31,129	0.8090	1.1000	No change
mmu-miR-31-5p	14,790	0.5746	19,956	0.5186	1.1078	No change
mmu-miR-125a-5p	42,440	1.6487	57,146	1.4852	1.1101	No change
mmu-miR-342-3p	20,643	0.8019	27,753	0.7213	1.1118	No change
mmu-miR-181a-1-3p	10,757	0.4179	14,459	0.3758	1.1120	No change
mmu-miR-338-5p	355	0.0138	476	0.0124	1.1148	No change
mmu-let-7a-5p	282,168	10.9615	378,095	9.8264	1.1155	No change
mmu-miR-296-5p	253	0.0098	339	0.0088	1.1155	No change
mmu-miR-6540-5p	3	0.000117	4	0.000104	1.1211	No change
mmu-miR-129-1-3p	3	0.000117	4	0.000104	1.1211	No change
mmu-miR-145b	3	0.000117	4	0.000104	1.1211	No change
mmu-miR-669p-3p	3	0.000117	4	0.000104	1.1211	No change
mmu-miR-3086-5p	6	0.000233	8	0.000208	1.1211	No change
mmu-miR-7017-5p	6	0.000233	8	0.000208	1.1211	No change
mmu-miR-297a-5p	6	0.000233	8	0.000208	1.1211	No change
mmu-miR-676-5p	314	0.0122	417	0.0108	1.1255	No change
mmu-miR-23b-3p	12,365	0.4803	16,414	0.4266	1.1260	No change
mmu-miR-5099	1,425	0.0554	1,891	0.0491	1.1264	No change
mmu-miR-27a-3p	92,899	3.6089	123,100	3.1993	1.1280	No change
mmu-miR-425-5p	16,417	0.6378	21,741	0.5650	1.1287	No change
mmu-miR-497-5p	1,012	0.0393	1,339	0.0348	1.1297	No change
mmu-miR-15a-3p	66	0.0026	87	0.0023	1.1339	No change
mmu-miR-5121	193	0.0075	254	0.0066	1.1358	No change
mmu-miR-125b-1-3p	23,407	0.9093	30,680	0.7974	1.1404	No change
mmu-miR-669o-5p	29	0.0011	38	0.0010	1.1407	No change
mmu-miR-24-3p	23,587	0.9163	30,680	0.7974	1.1492	No change
mmu-miR-106b-3p	4,465	0.1735	5,786	0.1504	1.1535	No change
mmu-miR-130b-3p	5,724	0.2224	7,409	0.1926	1.1548	No change
mmu-miR-5128	24	0.000932	31	0.000806	1.1572	No change
mmu-miR-196a-5p	8,010	0.3112	10,330	0.2685	1.1590	No change
mmu-miR-214-5p	1,855	0.0721	2,382	0.0619	1.1640	No change
mmu-miR-30c-5p	4,237	0.1646	5,427	0.1410	1.1670	No change
mmu-miR-27b-3p	2,424,527	94.1864	3,088,518	80.2686	1.1734	No change
mmu-miR-103-3p	116,214	4.5146	147,939	3.8448	1.1742	No change
mmu-miR-6970-5p	22	0.000855	28	0.000728	1.1744	No change
mmu-miR-484	7,349	0.2855	9,300	0.2417	1.1812	No change
mmu-miR-1249-3p	287	0.0111	363	0.0094	1.1818	No change
mmu-miR-7015-3p	156	0.0061	197	0.0051	1.1837	No change
mmu-miR-27b-5p	16,172	0.6282	20,379	0.5296	1.1862	No change
mmu-miR-7091-5p	144	0.0056	181	0.0047	1.1892	No change
mmu-miR-466p-3p	71	0.0028	89	0.0023	1.1924	No change
mmu-miR-466c-3p	71	0.0028	89	0.0023	1.1924	No change
mmu-miR-466b-3p	71	0.0028	89	0.0023	1.1924	No change
mmu-miR-351-5p	32,240	1.2524	40,394	1.0498	1.1930	No change
mmu-miR-702-3p	12	0.000466	15	0.000390	1.1958	No change
mmu-miR-188-5p	28	0.0011	35	0.000910	1.1958	No change
mmu-miR-652-5p	4	0.000155	5	0.000130	1.1958	No change

mmu-miR-181b-2-3p	4	0.000155	5	0.000130	1.1958	No change
mmu-miR-6999-5p	4	0.000155	5	0.000130	1.1958	No change
mmu-miR-1927	8	0.000311	10	0.000260	1.1958	No change
mmu-miR-466a-5p	8	0.000311	10	0.000260	1.1958	No change
mmu-miR-99b-3p	665	0.0258	831	0.0216	1.1962	No change
mmu-miR-28a-5p	25,798	1.0022	32,158	0.8358	1.1991	No change
mmu-miR-378b	171	0.0066	213	0.0055	1.2000	No change
mmu-miR-652-3p	4,453	0.1730	5,542	0.1440	1.2010	No change
mmu-miR-99b-5p	48,142	1.8702	59,670	1.5508	1.2060	No change
mmu-miR-1943-5p	1,100	0.0427	1,362	0.0354	1.2072	No change
mmu-miR-30c-2-3p	4,278	0.1662	5,257	0.1366	1.2164	No change
mmu-miR-324-3p	101	0.0039	124	0.0032	1.2175	No change
mmu-miR-7042-5p	18	0.000699	22	0.000572	1.2230	No change
mmu-miR-7043-3p	27	0.0010	33	0.000858	1.2230	No change
mmu-miR-3089-3p	32	0.0012	39	0.0010	1.2265	No change
mmu-miR-532-3p	1,342	0.0521	1,632	0.0424	1.2291	No change
mmu-miR-3076-3p	42	0.0016	51	0.0013	1.2310	No change
mmu-miR-28a-3p	7,659	0.2975	9,231	0.2399	1.2402	No change
mmu-miR-193b-5p	39	0.0015	47	0.0012	1.2403	No change
mmu-miR-708-3p	156	0.0061	188	0.0049	1.2403	No change
mmu-miR-182-5p	39,592	1.5380	47,653	1.2385	1.2419	No change
mmu-miR-532-5p	39,686	1.5417	47,745	1.2409	1.2424	No change
mmu-miR-199a-5p	6,995	0.2717	8,405	0.2184	1.2440	No change
mmu-let-7k	5	0.000194	6	0.000156	1.2456	No change
mmu-miR-370-3p	5	0.000194	6	0.000156	1.2456	No change
mmu-miR-6932-5p	5	0.000194	6	0.000156	1.2456	No change
mmu-miR-666-5p	15	0.000583	18	0.000468	1.2456	No change
mmu-miR-8105	26	0.0010	31	0.000806	1.2537	No change
mmu-miR-1934-3p	21	0.000816	25	0.000650	1.2556	No change
mmu-miR-6916-5p	102	0.0040	121	0.0031	1.2600	No change
mmu-miR-1191b-5p	161	0.0063	190	0.0049	1.2666	No change
mmu-miR-3074-1-3p	39	0.0015	46	0.0012	1.2673	No change
mmu-miR-1306-5p	117	0.0045	138	0.0036	1.2673	No change
mmu-miR-331-5p	96	0.0037	113	0.0029	1.2699	No change
mmu-miR-221-5p	6,773	0.2631	7,948	0.2066	1.2738	No change
mmu-miR-1231-3p	12	0.000466	14	0.000364	1.2812	No change
mmu-miR-7068-3p	129	0.0050	150	0.0039	1.2855	No change
mmu-miR-672-5p	56	0.0022	65	0.0017	1.2878	No change
mmu-miR-7237-3p	19	0.000738	22	0.000572	1.2909	No change
mmu-miR-6944-3p	170	0.0066	196	0.0051	1.2965	No change
mmu-miR-423-3p	41,446	1.6101	47,743	1.2408	1.2976	No change
mmu-miR-1947-5p	404	0.0157	465	0.0121	1.2987	No change
mmu-miR-431-3p	7	0.000272	8	0.000208	1.3079	No change
mmu-miR-802-3p	14	0.000544	16	0.000416	1.3079	No change
mmu-miR-345-3p	112	0.0044	128	0.0033	1.3079	No change
mmu-miR-664-3p	225	0.0087	257	0.0067	1.3086	No change
mmu-miR-3102-3p	253	0.0098	288	0.0075	1.3131	No change
mmu-miR-34c-3p	116	0.0045	132	0.0034	1.3136	No change
mmu-miR-100-5p	11,749	0.4564	13,327	0.3464	1.3178	No change
mmu-miR-669l-5p	62	0.0024	70	0.0018	1.3239	No change
mmu-miR-129-5p	1,735	0.0674	1,956	0.0508	1.3259	No change
mmu-miR-1981-3p	173	0.0067	195	0.0051	1.3261	No change
mmu-miR-8114	71	0.0028	80	0.0021	1.3266	No change
mmu-miR-298-5p	39,292	1.5264	44,104	1.1462	1.3317	No change
mmu-miR-700-3p	149	0.0058	167	0.0043	1.3336	No change
mmu-miR-125b-5p	72,989	2.8354	80,991	2.1049	1.3471	No change
mmu-miR-6966-3p	10	0.000388	11	0.000286	1.3589	No change
mmu-miR-5626-3p	10	0.000388	11	0.000286	1.3589	No change
mmu-miR-125a-3p	931	0.0362	1,020	0.0265	1.3643	No change
mmu-miR-7035-3p	129	0.0050	141	0.0037	1.3675	No change
mmu-miR-7084-5p	11	0.000427	12	0.000312	1.3702	No change
mmu-miR-17-3p	1,005	0.0390	1,095	0.0285	1.3719	No change
mmu-miR-615-5p	641	0.0249	697	0.0181	1.3746	No change
mmu-miR-99a-5p	24,297	0.9439	26,352	0.6849	1.3782	No change

mmu-miR-330-5p	216	0.0084	234	0.0061	1.3798	No change
mmu-miR-99a-3p	133	0.0052	144	0.0037	1.3806	No change
mmu-miR-18a-3p	85	0.0033	92	0.0024	1.3810	No change
mmu-let-7c-5p	335,005	13.0140	362,405	9.4187	1.3817	No change
mmu-miR-467d-3p	49	0.0019	53	0.0014	1.3819	No change
mmu-miR-191-5p	724,595	28.1486	782,310	20.3317	1.3845	No change
mmu-miR-224-5p	13	0.000505	14	0.000364	1.3880	No change
mmu-miR-149-5p	6,413	0.2491	6,886	0.1790	1.3921	No change
mmu-miR-501-3p	4,454	0.1730	4,769	0.1239	1.3960	No change
mmu-miR-1198-5p	2,073	0.0805	2,216	0.0576	1.3983	No change
mmu-miR-326-5p	29	0.0011	31	0.000806	1.3983	No change
mmu-miR-7648-3p	30	0.0012	32	0.000832	1.4013	No change
mmu-miR-669b-5p	16	0.000622	17	0.000442	1.4068	No change
mmu-let-7d-3p	29,937	1.1630	31,751	0.8252	1.4093	No change
mmu-miR-30a-3p	8,893	0.3455	9,426	0.2450	1.4102	No change
mmu-miR-6986-5p	34	0.0013	36	0.000936	1.4117	No change
mmu-miR-378c	19,776	0.7682	20,936	0.5441	1.4119	No change
mmu-miR-7687-5p	118	0.0046	124	0.0032	1.4224	No change
mmu-miR-326-3p	2,028	0.0788	2,128	0.0553	1.4245	No change
mmu-miR-423-5p	231,109	8.9780	241,757	6.2831	1.4289	No change
mmu-miR-204-5p	67	0.0026	70	0.0018	1.4307	No change
mmu-miR-542-5p	190	0.0074	197	0.0051	1.4416	No change
mmu-miR-574-3p	3,780	0.1468	3,916	0.1018	1.4428	No change
mmu-miR-183-5p	1,153	0.0448	1,192	0.0310	1.4458	No change
mmu-miR-378a-5p	488	0.0190	504	0.0131	1.4473	No change
mmu-miR-7086-5p	66	0.0026	68	0.0018	1.4508	No change
mmu-miR-324-5p	1,034	0.0402	1,063	0.0276	1.4540	No change
mmu-miR-425-3p	255	0.0099	262	0.0068	1.4548	No change
mmu-miR-185-3p	728	0.0283	747	0.0194	1.4567	No change
mmu-miR-139-5p	310	0.0120	318	0.0083	1.4571	No change
mmu-miR-378a-3p	145,985	5.6711	149,442	3.8839	1.4602	No change
mmu-miR-296-3p	4,221	0.1640	4,298	0.1117	1.4680	No change
mmu-miR-7688-5p	2,261	0.0878	2,293	0.0596	1.4739	No change
mmu-miR-744-5p	126,495	4.9140	127,713	3.3192	1.4805	No change
mmu-miR-30e-3p	15,677	0.6090	15,730	0.4088	1.4897	No change
mmu-miR-92b-3p	1,634	0.0635	1,636	0.0425	1.4929	No change
mmu-miR-3062-5p	3	0.000117	3	0.000078	1.4947	No change
mmu-miR-6953-3p	3	0.000117	3	0.000078	1.4947	No change
mmu-miR-7687-3p	3	0.000117	3	0.000078	1.4947	No change
mmu-miR-7665-5p	3	0.000117	3	0.000078	1.4947	No change
mmu-miR-130a-5p	6	0.000233	6	0.000156	1.4947	No change
mmu-miR-874-5p	12	0.000466	12	0.000312	1.4947	No change
mmu-miR-466i-3p	17	0.000660	17	0.000442	1.4947	No change
mmu-miR-485-5p	28	0.001088	28	0.000728	1.4947	No change
mmu-miR-5617-3p	1	0.000039	1	0.000026	1.4947	No change
mmu-miR-669h-5p	1	0.000039	1	0.000026	1.4947	No change
mmu-miR-9-3p	1	0.000039	1	0.000026	1.4947	No change
mmu-miR-1668	1	0.000039	1	0.000026	1.4947	No change
mmu-miR-7647-3p	1	0.000039	1	0.000026	1.4947	No change
mmu-miR-21c	1	0.000039	1	0.000026	1.4947	No change
mmu-miR-341-5p	1	0.000039	1	0.000026	1.4947	No change
mmu-miR-133b-3p	2	0.000078	2	0.000052	1.4947	No change
mmu-miR-376a-5p	2	0.000078	2	0.000052	1.4947	No change
mmu-miR-499-5p	2	0.000078	2	0.000052	1.4947	No change
mmu-miR-190b-3p	2	0.000078	2	0.000052	1.4947	No change
mmu-miR-6418-3p	2	0.000078	2	0.000052	1.4947	No change
mmu-miR-873a-5p	2	0.000078	2	0.000052	1.4947	No change
mmu-miR-1946b	2	0.000078	2	0.000052	1.4947	No change
mmu-miR-6929-3p	2	0.000078	2	0.000052	1.4947	No change
mmu-miR-190a-3p	2	0.000078	2	0.000052	1.4947	No change
mmu-miR-675-3p	4	0.000155	4	0.000104	1.4947	No change
mmu-miR-3077-3p	4	0.000155	4	0.000104	1.4947	No change
mmu-miR-143-5p	4	0.000155	4	0.000104	1.4947	No change
mmu-miR-92b-5p	4	0.000155	4	0.000104	1.4947	No change

mmu-miR-6908-3p	5	0.000194	5	0.000130	1.4947	No change
mmu-miR-466p-5p	8	0.000311	8	0.000208	1.4947	No change
mmu-miR-101c	11	0.000427	11	0.000286	1.4947	No change
mmu-miR-33-3p	41	0.0016	41	0.0011	1.4947	No change
mmu-miR-466a-3p	141	0.0055	141	0.0037	1.4947	No change
mmu-miR-466e-3p	141	0.0055	141	0.0037	1.4947	No change
mmu-miR-339-5p	12,100	0.4701	11,915	0.3097	1.5179	Up
mmu-miR-151-3p	78,623	3.0543	77,302	2.0090	1.5203	Up
mmu-miR-6948-3p	94	0.0037	92	0.0024	1.5272	No change
mmu-miR-29b-2-5p	83	0.0032	81	0.0021	1.5316	No change
mmu-miR-218-1-3p	206	0.0080	201	0.0052	1.5319	Up
mmu-miR-130b-5p	1,452	0.0564	1,413	0.0367	1.5360	Up
mmu-miR-3098-5p	29	0.001127	28	0.000728	1.5481	No change
mmu-miR-365-2-5p	141	0.0055	136	0.0035	1.5497	No change
mmu-miR-708-5p	48	0.0019	46	0.0012	1.5597	No change
mmu-miR-193b-3p	600	0.0233	574	0.0149	1.5624	Up
mmu-miR-1960	69	0.0027	66	0.0017	1.5627	No change
mmu-miR-152-3p	738	0.0287	702	0.0182	1.5714	Up
mmu-miR-30c-1-3p	3,238	0.1258	3,067	0.0797	1.5781	Up
mmu-miR-328-5p	33	0.0013	31	0.000806	1.5912	No change
mmu-miR-15b-5p	13,487	0.5239	12,606	0.3276	1.5992	Up
mmu-miR-676-3p	11,226	0.4361	10,487	0.2726	1.6001	Up
mmu-miR-700-5p	58	0.0023	54	0.0014	1.6055	No change
mmu-miR-3075-5p	25	0.000971	23	0.000598	1.6247	No change
mmu-miR-351-3p	25	0.000971	23	0.000598	1.6247	No change
mmu-miR-146b-3p	61	0.0024	56	0.0015	1.6282	No change
mmu-miR-6899-3p	24	0.000932	22	0.000572	1.6306	No change
mmu-miR-877-5p	619	0.0240	566	0.0147	1.6347	Up
mmu-miR-7012-5p	138	0.0054	126	0.0033	1.6371	No change
mmu-miR-339-3p	3,313	0.1287	3,013	0.0783	1.6436	Up
mmu-miR-181a-5p	1,986,363	77.1649	1,802,168	46.8372	1.6475	Up
mmu-miR-382-5p	10	0.000388	9	0.000234	1.6608	No change
mmu-miR-500-3p	1,219	0.0474	1,086	0.0282	1.6778	Up
mmu-miR-3066-3p	9	0.000350	8	0.000208	1.6816	No change
mmu-miR-1199-5p	1,000	0.0388	875	0.0227	1.7083	Up
mmu-miR-669a-3p	141	0.0055	123	0.0032	1.7135	No change
mmu-miR-669o-3p	141	0.0055	123	0.0032	1.7135	No change
mmu-miR-140-3p	3,166	0.1230	2,757	0.0717	1.7165	Up
mmu-miR-345-5p	373	0.0145	322	0.0084	1.7315	Up
mmu-miR-7063-5p	63	0.0024	54	0.0014	1.7439	No change
mmu-miR-505-5p	105	0.0041	90	0.0023	1.7439	No change
mmu-miR-320-3p	9,565	0.3716	8,123	0.2111	1.7601	Up
mmu-miR-3057-5p	3,850	0.1496	3,267	0.0849	1.7615	Up
mmu-miR-331-3p	490	0.0190	412	0.0107	1.7777	Up
mmu-miR-3060-3p	6	0.000233	5	0.000130	1.7937	No change
mmu-miR-214-3p	5,756	0.2236	4,785	0.1244	1.7981	Up
mmu-miR-5119	59	0.0023	49	0.0013	1.7998	No change
mmu-miR-30f	813	0.0316	675	0.0175	1.8003	Up
mmu-let-7i-3p	629	0.0244	519	0.0135	1.8115	Up
mmu-miR-466d-5p	17	0.000660	14	0.000364	1.8150	No change
mmu-miR-328-3p	7,760	0.3015	6,367	0.1655	1.8218	Up
mmu-miR-1932	11	0.000427	9	0.000234	1.8269	No change
mmu-miR-34a-3p	11	0.000427	9	0.000234	1.8269	No change
mmu-miR-879-5p	11	0.000427	9	0.000234	1.8269	No change
mmu-let-7b-5p	27,852	1.0820	22,737	0.5909	1.8310	Up
mmu-miR-7093-3p	16	0.000622	13	0.000338	1.8397	No change
mmu-miR-3102-5p.2-5p	225	0.0087	182	0.0047	1.8479	No change
mmu-miR-6997-5p	31	0.001204	25	0.000650	1.8535	No change
mmu-miR-6516-5p	46	0.0018	37	0.0010	1.8583	No change
mmu-miR-10b-3p	5	0.000194	4	0.000104	1.8684	No change
mmu-miR-196b-3p	5	0.000194	4	0.000104	1.8684	No change
mmu-miR-466n-5p	10	0.000388	8	0.000208	1.8684	No change
mmu-miR-3069-5p	10	0.000388	8	0.000208	1.8684	No change
mmu-miR-1930-5p	49	0.0019	39	0.0010	1.8780	No change

mmu-miR-185-5p	52	0.0020	41	0.0011	1.8958	No change
mmu-let-7b-3p	193	0.0075	152	0.0040	1.8979	No change
mmu-miR-3064-5p	125	0.0049	98	0.0025	1.9066	No change
mmu-miR-671-5p	9	0.000350	7	0.000182	1.9218	No change
mmu-miR-455-3p	21	0.000816	16	0.000416	1.9618	No change
mmu-miR-664-5p	310	0.0120	236	0.0061	1.9634	Up
mmu-miR-365-1-5p	29	0.001127	22	0.000572	1.9703	No change
mmu-miR-466b-5p	8	0.000311	6	0.000156	1.9930	No change
mmu-miR-466o-5p	8	0.000311	6	0.000156	1.9930	No change
mmu-miR-7679-3p	8	0.000311	6	0.000156	1.9930	No change
mmu-miR-500-5p	12	0.000466	9	0.000234	1.9930	No change
mmu-miR-6236	24	0.000932	18	0.000468	1.9930	No change
mmu-miR-146a-3p	24	0.000932	18	0.000468	1.9930	No change
mmu-miR-6990-5p	51	0.0020	38	0.0010	2.0061	No change
mmu-miR-690	121	0.0047	90	0.0023	2.0096	No change
mmu-miR-467a-3p	35	0.001360	26	0.000676	2.0121	No change
mmu-miR-30b-3p	876	0.0340	640	0.0166	2.0459	Up
mmu-miR-148b-3p	5,837	0.2268	4,252	0.1105	2.0519	Up
mmu-miR-210-3p	20,314	0.7891	14,712	0.3824	2.0639	Up
mmu-miR-7031-5p	7	0.000272	5	0.000130	2.0926	No change
mmu-miR-1843a-3p	470	0.0183	334	0.0087	2.1034	Up
mmu-miR-203-3p	1,142	0.0444	809	0.0210	2.1100	Up
mmu-miR-25-5p	357	0.0139	252	0.0065	2.1175	Up
mmu-miR-487b-3p	10	0.000388	7	0.000182	2.1353	No change
mmu-miR-330-3p	188	0.0073	131	0.0034	2.1451	No change
mmu-miR-6909-5p	23	0.000893	16	0.000416	2.1487	No change
mmu-miR-7013-5p	13	0.000505	9	0.000234	2.1591	No change
mmu-miR-7671-3p	35	0.001360	24	0.000624	2.1798	No change
mmu-miR-574-5p	1,408	0.0547	957	0.0249	2.1992	Up
mmu-miR-874-3p	31	0.001204	21	0.000546	2.2065	No change
mmu-miR-674-5p	548	0.0213	368	0.0096	2.2259	Up
mmu-miR-511-5p	18	0.000699	12	0.000312	2.2421	No change
mmu-miR-450a-1-3p	3	0.000117	2	0.000052	2.2421	No change
mmu-miR-7030-5p	3	0.000117	2	0.000052	2.2421	No change
mmu-miR-7062-3p	3	0.000117	2	0.000052	2.2421	No change
mmu-miR-7674-3p	3	0.000117	2	0.000052	2.2421	No change
mmu-miR-7081-5p	3	0.000117	2	0.000052	2.2421	No change
mmu-miR-6942-5p	3	0.000117	2	0.000052	2.2421	No change
mmu-miR-1946a	3	0.000117	2	0.000052	2.2421	No change
mmu-miR-7060-5p	6	0.000233	4	0.000104	2.2421	No change
mmu-miR-7009-5p	83	0.0032	55	0.0014	2.2557	No change
mmu-miR-27a-5p	6,948	0.2699	4,598	0.1195	2.2587	Up
mmu-miR-504-5p	44	0.001709	29	0.000754	2.2679	No change
mmu-miR-7083-5p	29	0.001127	19	0.000494	2.2814	No change
mmu-miR-205-5p	99	0.0038	64	0.0017	2.3122	No change
mmu-miR-7013-3p	11	0.000427	7	0.000182	2.3489	No change
mmu-miR-503-3p	43	0.001670	27	0.000702	2.3805	No change
mmu-miR-5114	110	0.0043	69	0.0018	2.3829	No change
mmu-miR-5709-5p	8	0.000311	5	0.000130	2.3916	No change
mmu-miR-7674-5p	29	0.001127	18	0.000468	2.4082	No change
mmu-miR-134-5p	100	0.0039	61	0.0016	2.4504	No change
mmu-miR-1306-3p	53	0.0021	32	0.000832	2.4757	No change
mmu-miR-7025-3p	5	0.000194	3	0.000078	2.4912	No change
mmu-miR-205-3p	5	0.000194	3	0.000078	2.4912	No change
mmu-miR-7656-3p	5	0.000194	3	0.000078	2.4912	No change
mmu-miR-669f-3p	22	0.000855	13	0.000338	2.5296	No change
mmu-miR-6952-5p	24	0.000932	14	0.000364	2.5624	No change
mmu-miR-200c-3p	24	0.000932	14	0.000364	2.5624	No change
mmu-miR-483-5p	7	0.000272	4	0.000104	2.6158	No change
mmu-miR-7672-3p	7	0.000272	4	0.000104	2.6158	No change
mmu-miR-7676-3p	51	0.001981	29	0.000754	2.6287	No change
mmu-miR-200a-5p	9	0.000350	5	0.000130	2.6905	No change
mmu-miR-6395	38	0.001476	21	0.000546	2.7048	No change
mmu-miR-1981-5p	2,582	0.1003	1,394	0.0362	2.7686	Up

mmu-miR-598-3p	71	0.0028	38	0.0010	2.7928	No change
mmu-miR-92a-1-5p	5,832	0.2266	3,119	0.0811	2.7949	Up
mmu-miR-7656-5p	6	0.000233	3	0.000078	2.9895	No change
mmu-miR-3072-5p	6	0.000233	3	0.000078	2.9895	No change
mmu-miR-466d-3p	14	0.000544	7	0.000182	2.9895	No change
mmu-miR-5620-5p	24	0.000932	12	0.000312	2.9895	No change
mmu-miR-494-3p	2	0.000078	1	0.000026	2.9895	No change
mmu-miR-540-5p	2	0.000078	1	0.000026	2.9895	No change
mmu-miR-6963-5p	2	0.000078	1	0.000026	2.9895	No change
mmu-miR-7689-3p	2	0.000078	1	0.000026	2.9895	No change
mmu-miR-3083-5p	2	0.000078	1	0.000026	2.9895	No change
mmu-miR-7231-5p	2	0.000078	1	0.000026	2.9895	No change
mmu-miR-433-5p	2	0.000078	1	0.000026	2.9895	No change
mmu-miR-6983-5p	2	0.000078	1	0.000026	2.9895	No change
mmu-miR-668-3p	4	0.000155	2	0.000052	2.9895	No change
mmu-miR-421-5p	4	0.000155	2	0.000052	2.9895	No change
mmu-miR-6946-5p	4	0.000155	2	0.000052	2.9895	No change
mmu-miR-6956-5p	4	0.000155	2	0.000052	2.9895	No change
mmu-miR-5134-3p	4	0.000155	2	0.000052	2.9895	No change
mmu-miR-6984-5p	8	0.000311	4	0.000104	2.9895	No change
mmu-miR-3084-3p	17	0.000660	8	0.000208	3.1763	No change
mmu-miR-194-2-3p	45	0.001748	21	0.000546	3.2030	No change
mmu-miR-375-3p	48	0.001865	22	0.000572	3.2612	No change
mmu-miR-7082-3p	11	0.000427	5	0.000130	3.2884	No change
mmu-miR-5129-3p	11	0.000427	5	0.000130	3.2884	No change
mmu-miR-7118-3p	9	0.000350	4	0.000104	3.3632	No change
mmu-miR-141-5p	7	0.000272	3	0.000078	3.4877	No change
mmu-miR-1958	12	0.000466	5	0.000130	3.5874	No change
mmu-miR-1195	272	0.0106	111	0.0029	3.6628	No change
mmu-miR-155-5p	1,052	0.0409	421	0.0109	3.7351	Up
mmu-miR-7068-5p	5	0.000194	2	0.000052	3.7368	No change
mmu-miR-6921-5p	5	0.000194	2	0.000052	3.7368	No change
mmu-miR-29b-1-5p	5	0.000194	2	0.000052	3.7368	No change
mmu-miR-8098	5	0.000194	2	0.000052	3.7368	No change
mmu-miR-7014-5p	10	0.000388	4	0.000104	3.7368	No change
mmu-miR-5129-5p	8	0.000311	3	0.000078	3.9860	No change
mmu-miR-1954	11	0.000427	4	0.000104	4.1105	No change
mmu-miR-6911-3p	28	0.001088	10	0.000260	4.1853	No change
mmu-miR-23b-5p	17	0.000660	6	0.000156	4.2351	No change
mmu-miR-6985-5p	9	0.000350	3	0.000078	4.4842	No change
mmu-miR-181a-2-3p	9	0.000350	3	0.000078	4.4842	No change
mmu-miR-295-3p	3	0.000117	1	0.000026	4.4842	No change
mmu-miR-466e-5p	3	0.000117	1	0.000026	4.4842	No change
mmu-miR-103-1-5p	3	0.000117	1	0.000026	4.4842	No change
mmu-miR-3470b	89	0.003457	28	0.000728	4.7511	No change
mmu-miR-3470a	74	0.002875	23	0.000598	4.8092	No change
mmu-miR-6922-5p	13	0.000505	4	0.000104	4.8579	No change
mmu-miR-3473g	10	0.000388	3	0.000078	4.9825	No change
mmu-miR-7062-5p	12	0.000466	3	0.000078	5.9790	No change
mmu-miR-5620-3p	48	0.001865	12	0.000312	5.9790	No change
mmu-miR-7655-3p	4	0.000155	1	0.000026	5.9790	No change
mmu-miR-5126	89	0.003457	21	0.000546	6.3348	No change
mmu-miR-7087-5p	13	0.000505	3	0.000078	6.4772	No change
mmu-miR-7668-3p	13	0.000505	3	0.000078	6.4772	No change
mmu-miR-6966-5p	9	0.000350	2	0.000052	6.7263	No change
mmu-miR-5627-3p	23	0.000893	5	0.000130	6.8758	No change
mmu-miR-7024-3p	5	0.000194	1	0.000026	7.4737	No change
mmu-miR-7667-5p	5	0.000194	1	0.000026	7.4737	No change
mmu-miR-192-3p	5	0.000194	1	0.000026	7.4737	No change
mmu-miR-6978-3p	10	0.000388	2	0.000052	7.4737	No change
mmu-miR-7654-5p	10	0.000388	2	0.000052	7.4737	No change
mmu-miR-7015-5p	10	0.000388	2	0.000052	7.4737	No change
mmu-miR-503-5p	56	0.002175	11	0.000286	7.6096	No change
mmu-miR-6538	204	0.0079	40	0.0010	7.6232	No change

mmu-miR-669c-5p	26	0.001010	5	0.000130	7.7726	No change
mmu-miR-6905-5p	11	0.000427	2	0.000052	8.2211	No change
mmu-miR-7117-5p	6	0.000233	1	0.000026	8.9684	No change
mmu-miR-5130	19	0.000738	3	0.000078	9.4667	No change
mmu-miR-1941-5p	13	0.000505	2	0.000052	9.7158	No change
mmu-miR-7046-5p	7	0.000272	1	0.000026	10.4632	No change
mmu-miR-5107-5p	7	0.000272	1	0.000026	10.4632	No change
mmu-miR-7655-5p	7	0.000272	1	0.000026	10.4632	No change
mmu-miR-1936	7	0.000272	1	0.000026	10.4632	No change
mmu-miR-6240	4,731	0.1838	635	0.0165	11.1364	Up
mmu-miR-7669-3p	8	0.000311	1	0.000026	11.9579	No change
mmu-miR-1940	589	0.0229	53	0.0014	16.6113	No change
mmu-miR-155-3p	35	0.001360	2	0.000052	26.1579	No change

miRNAs in red show no differential expression between infected and control macrophages or are below the 200 reads per sample expression. miRNAs in grey show differential expression between infected and control macrophages. Green shading shows all miRNAs with more than 1.5 fold higher expression in control macrophages. Red shading shows all miRNAs with more than 1.5 fold higher expression in infected macrophages.
