# Supplementary material for: miR-181a-5p Regulates TNF-α and miR-21a-5p Influences Gualynate-Binding Protein 5 and IL-10 Expression in Macrophages Affecting Host Control of Brucella abortus Infection
Source: Front Immunol. 2018 Jun 11;9:1331. doi: 10.3389/fimmu.2018.01331 (PMC6004377; doi:10.3389/fimmu.2018.01331)
Supplement: Supplementary file 3 [file Table_3.PDF]

Supplementary Table 3. Immune system-related mRNA targets for nine miRNAs differentially expressed during *B. abortus* infection.

Target Genes *	miRNA
**Interferon Signaling** *R-MMU-913531#*	
Eif4a2	mmu-miR-181a-5p
Eif4g3	mmu-miR-181a-5p
Gbp7	mmu-miR-181a-5p
Irf4	mmu-miR-181a-5p
Kpna4	mmu-miR-181a-5p
Mid1	mmu-miR-181a-5p
Nup214	mmu-miR-181a-5p
Ranbp2	mmu-miR-181a-5p
Tpr	mmu-miR-181a-5p
Gbp2	mmu-miR-21a-5p
Gbp2b	mmu-miR-21a-5p
Gbp4	mmu-miR-21a-5p
**Gbp5**	**mmu-miR-21a-5p**
Gbp8	mmu-miR-21a-5p
Abce1	mmu-miR-21a-5p
Camk2d	mmu-miR-21a-5p
Eif4e2	mmu-miR-21a-5p
Eif4g3	mmu-miR-21a-5p
Ncam1	mmu-miR-21a-5p
Nup205	mmu-miR-21a-5p
Nup93	mmu-miR-21a-5p
Nup98	mmu-miR-21a-5p
Ptpn2	mmu-miR-21a-5p
Stat1	mmu-miR-21a-5p
Trim46	mmu-miR-21a-5p
Camk2a	mmu-miR-145a-3p
Eif4e2	mmu-miR-145a-3p
Eif4g1	mmu-miR-145a-3p
Eif4g3	mmu-miR-145a-3p
Gbp7	mmu-miR-145a-3p
Isg15	mmu-miR-145a-3p
Ncam1	mmu-miR-145a-3p
Nup188	mmu-miR-145a-3p
Oas2	mmu-miR-145a-3p
Stat2	mmu-miR-145a-3p
Sumo1	mmu-miR-145a-3p
Trim14	mmu-miR-145a-3p
Trim45	mmu-miR-145a-3p
Eif4e2	mmu-miR-146b-5p
Gbp7	mmu-miR-146b-5p
Ifit2	mmu-miR-146b-5p

Irf8	mmu-miR-146b-5p
Kpna4	mmu-miR-146b-5p
Nedd4	mmu-miR-146b-5p
Nup107	mmu-miR-146b-5p
Nup133	mmu-miR-146b-5p
Nup155	mmu-miR-146b-5p
Rsad2	mmu-miR-146b-5p
Stat2	mmu-miR-146b-5p
Sumo1	mmu-miR-146b-5p
Trim14	mmu-miR-146b-5p
Trim5	mmu-miR-146b-5p
Uba7	mmu-miR-146b-5p
Camk2a	mmu-miR-151-3p
Camk2b	mmu-miR-151-3p
Ddx58	mmu-miR-151-3p
Eif4a1	mmu-miR-151-3p
Eif4e	mmu-miR-151-3p
Eif4e2	mmu-miR-151-3p
Eif4g1	mmu-miR-151-3p
Flnb	mmu-miR-151-3p
Ifit1	mmu-miR-151-3p
Ifngr2	mmu-miR-151-3p
Irf1	mmu-miR-151-3p
Irf3	mmu-miR-151-3p
Irf4	mmu-miR-151-3p
Isg15	mmu-miR-151-3p
Kpna1	mmu-miR-151-3p
Kpnb1	mmu-miR-151-3p
Ncam1	mmu-miR-151-3p
Nup160	mmu-miR-151-3p
Nup205	mmu-miR-151-3p
Nup214	mmu-miR-151-3p
Nup50	mmu-miR-151-3p
Sumo1	mmu-miR-151-3p
Trim21	mmu-miR-151-3p
Trim3	mmu-miR-151-3p
Trim38	mmu-miR-151-3p
Trim6	mmu-miR-151-3p
Trim68	mmu-miR-151-3p
Trim5	mmu-miR-155-5p
Camk2a	mmu-miR-328-3p
Eif4a2	mmu-miR-328-3p
Eif4e3	mmu-miR-328-3p
Eif4g1	mmu-miR-328-3p
Eif4g2	mmu-miR-328-3p
Gbp7	mmu-miR-328-3p

Ifnar1	mmu-miR-328-3p
Ip6k2	mmu-miR-328-3p
Irf1	mmu-miR-328-3p
Irf2	mmu-miR-328-3p
Irf4	mmu-miR-328-3p
Irf7	mmu-miR-328-3p
Jak1	mmu-miR-328-3p
Ncam1	mmu-miR-328-3p
Nup160	mmu-miR-328-3p
Nup205	mmu-miR-328-3p
Nup214	mmu-miR-328-3p
Nup35	mmu-miR-328-3p
Nup37	mmu-miR-328-3p
Nup98	mmu-miR-328-3p
Oas2	mmu-miR-328-3p
Pin1	mmu-miR-328-3p
Pml	mmu-miR-328-3p
Prkcd	mmu-miR-328-3p
Trim14	mmu-miR-328-3p
Trim38	mmu-miR-328-3p
Trim45	mmu-miR-328-3p
Trim62	mmu-miR-328-3p
Vcam1	mmu-miR-328-3p
Xaf1	mmu-miR-328-3p
Psmb8	mmu-miR-374b-5p
Arih1	mmu-miR-98-5p
Gbp7	mmu-miR-98-5p
Irf9	mmu-miR-98-5p
Kpna7	mmu-miR-98-5p
Nup107	mmu-miR-98-5p
Nup188	mmu-miR-98-5p
Nup210	mmu-miR-98-5p
Nup214	mmu-miR-98-5p
Nup37	mmu-miR-98-5p
Nup62	mmu-miR-98-5p
Pom121	mmu-miR-98-5p
Prkcd	mmu-miR-98-5p
Ptpn1	mmu-miR-98-5p
Ptpn2	mmu-miR-98-5p
Rae1	mmu-miR-98-5p
Rps27a	mmu-miR-98-5p
Sp100	mmu-miR-98-5p
Trim2	mmu-miR-98-5p
Trim6	mmu-miR-98-5p

Interleukin-10 signaling

R-MMU-6783783#

Csf3	mmu-miR-181a-5p
Il1a	mmu-miR-181a-5p
Ccr2	mmu-miR-21a-5p
Ccr2	mmu-miR-145a-3p
Il1r2	mmu-miR-145a-3p
Tnfrsf1b	mmu-miR-145a-3p
Il10ra	mmu-miR-146b-5p
Il1rn	mmu-miR-146b-5p
Stat3	mmu-miR-146b-5p
Ccr5	mmu-miR-151-3p
Il10ra	mmu-miR-151-3p
Il1b	mmu-miR-151-3p
Stat3	mmu-miR-151-3p
Tnf	mmu-miR-151-3p
Ccr2	mmu-miR-328-3p
Fpr1	mmu-miR-328-3p
Il1rn	mmu-miR-328-3p
Jak1	mmu-miR-328-3p
Lif	mmu-miR-328-3p
Fpr1	mmu-miR-98-5p
Il10rb	mmu-miR-98-5p
Il12b	mmu-miR-98-5p
Il13	mmu-miR-98-5p
Il1a	mmu-miR-98-5p
Il1r1	mmu-miR-98-5p
Tnf	mmu-miR-98-5p

Interleukin-12 family signaling
R-MMU-447115#

**Pdcd4**	**mmu-miR-21a-5p**
Foxo3	mmu-miR-21a-5p
Il12rb2	mmu-miR-21a-5p
Il27ra	mmu-miR-21a-5p
Lcp1	mmu-miR-21a-5p
Stat1	mmu-miR-21a-5p
Anxa2	mmu-miR-145a-3p
Hnrnpf	mmu-miR-145a-3p
Lmnb1	mmu-miR-145a-3p
Rala	mmu-miR-145a-3p
Pak2	mmu-miR-146b-5p
Stat3	mmu-miR-146b-5p
Stat3	mmu-miR-151-3p
Hnrnpf	mmu-miR-155-5p
Anxa2	mmu-miR-328-3p

Canx	mmu-miR-328-3p
Cnn2	mmu-miR-328-3p
Jak1	mmu-miR-328-3p
Pitpna	mmu-miR-328-3p
Serpinb2	mmu-miR-328-3p
Snrpa1	mmu-miR-328-3p
Il12b	mmu-miR-98-5p
Il12rb2	mmu-miR-98-5p
Sod2	mmu-miR-98-5p
**Regulation of TNFR1 signaling** *R-MMU-5357905*[#]	
**Tnf**	**mmu-miR-181a-5p**
Ikbkb	mmu-miR-181a-5p
Otud7b	mmu-miR-181a-5p
Rbck1	mmu-miR-181a-5p
Sppl2b	mmu-miR-181a-5p
Birc3	mmu-miR-21a-5p
Chuk	mmu-miR-21a-5p
Sppl2a	mmu-miR-21a-5p
Ikbkg	mmu-miR-145a-3p
Ripk1	mmu-miR-146b-5p
Tnfaip3	mmu-miR-146b-5p
Usp2	mmu-miR-146b-5p
Ikbkb	mmu-miR-151-3p
Madd	mmu-miR-151-3p
Otud7b	mmu-miR-151-3p
Sharpin	mmu-miR-151-3p
Tnf	mmu-miR-151-3p
Otud7b	mmu-miR-328-3p
Tnfaip3	mmu-miR-328-3p
Traf1	mmu-miR-328-3p
Traf2	mmu-miR-328-3p
Usp2	mmu-miR-328-3p
Usp21	mmu-miR-328-3p
Xiap	mmu-miR-328-3p
Birc3	mmu-miR-98-5p
Cyld	mmu-miR-98-5p
Ikbkb	mmu-miR-98-5p
Rps27a	mmu-miR-98-5p
Tax1bp1	mmu-miR-98-5p
Tnf	mmu-miR-98-5p
Tnfaip3	mmu-miR-98-5p

*Targets analyzed in this work are highlighted

[#] Reactome code for indicated pathways
