# Supplementary material for: miR-181a-5p Regulates TNF-α and miR-21a-5p Influences Gualynate-Binding Protein 5 and IL-10 Expression in Macrophages Affecting Host Control of Brucella abortus Infection
Source: Front Immunol. 2018 Jun 11;9:1331. doi: 10.3389/fimmu.2018.01331 (PMC6004377; doi:10.3389/fimmu.2018.01331)
Supplement: Supplementary file 4 [file Table_4.PDF]

Supplementary Table 4. Size of small RNAs from non-infected BMDM (GUT 8) or BMDMs 30-minutes after *B. abortus* infection (GUT 6) library.

**Insert range**		**0**	**1-17**	**18-26**	**27-44**
**GUT-6**	reads	204,080	5,658,079	31,332,945	1,318,775
	% reads	0.52	14.50	80.29	3.38
	%inserts	0.53	14.69	81.35	3.42
**GUT-8**	reads	77,431	4,043,972	43,451,966	1,089,676
	% reads	0.16	8.20	88.08	2.21
	%inserts	0.16	8.31	89.29	2.24
