# Supplementary material for: miR-181a-5p Regulates TNF-α and miR-21a-5p Influences Gualynate-Binding Protein 5 and IL-10 Expression in Macrophages Affecting Host Control of Brucella abortus Infection
Source: Front Immunol. 2018 Jun 11;9:1331. doi: 10.3389/fimmu.2018.01331 (PMC6004377; doi:10.3389/fimmu.2018.01331)
Supplement: Supplementary file 5 [file Table_5.PDF]

Supplementary Table 5. Overview of sequencing results

	***B. abortus* Infected BMDMs**	**%**	**Control non-infected BMDMs**	**% of total**
Processed *Reads*	35,800,946		46,518,441	
Mapped *Reads*	27,974,937	78.14% of the total	38,497,994	82.76% of the total
Reads mapped in the *B. abortus* (S2308) genome	2,077,235	7.43% of the total	13,257	0.03% of the total
Reads mapped in the *Mus musculus* (GRCm38) genome	25,741,795	92.02% of mapped reads	38,477,277	99.95% of mapped reads
Number of *reads* derived from miRNAs	21,165,237	81.73% of mapped reads	34,473,771	89.58% of mapped reads
Number of miRNAs identified	800		819	
Number of miRNAs identified in both libraries	745		745	
Number of *reads* derived from mRNAs	1,083,711	4.185% of mapped reads	1,047,898	2.723% of mapped reads
Number of *reads* derived from tRNAs	802,185	3.098% of mapped reads	1,021,095	2.653% of mapped reads
Number of *reads* derived from snRNAs	30,217	0.117% of mapped reads	28,557	0.074% of mapped reads
Number of *reads* derived from snoRNAs	248,679	0.960% of mapped reads	365,018	0.948% of mapped reads
Number of *reads* derived from rRNAs	354,937	1.371% of mapped reads	814,186	2.116% of mapped reads
