# Supplementary material for: miR-181a-5p Regulates TNF-α and miR-21a-5p Influences Gualynate-Binding Protein 5 and IL-10 Expression in Macrophages Affecting Host Control of Brucella abortus Infection
Source: Front Immunol. 2018 Jun 11;9:1331. doi: 10.3389/fimmu.2018.01331 (PMC6004377; doi:10.3389/fimmu.2018.01331)
Supplement: Supplementary file 6 [file Table_6.PDF]

Supplementary Table 6- Sequence of validated miRNAs used in this study

**miRNA**	**Mature sequence**	**Accession number***
mmu-miR-151-3p	CUAGACUGAGGCUCCUUGAGG	MIMAT0000161
mmu-miR-155-5p	UUAAUGCUAAUUGUGAUAGGGGU	MIMAT0000165
mmu-miR-181a-5p	AACAUUCAACGCUGUCGGUGAGU	MIMAT0000210
mmu-miR-328-3p	CUGGCCCUCUCUGCCCUUCCGU	MIMAT0000565
mmu-miR-21a-5p	UAGCUUAUCAGACUGAUGUUGA	MIMAT0000530
mmu-miR-98-5p	UGAGGUAGUAAGUUGUAUUGUU	MIMAT0000545
mmu-miR-145a-3p	AUUCCUGGAAAUACUGUUCUUG	MIMAT0004534
mmu-miR-146b-5p	UGAGAACUGAAUUCCAUAGGCU	MIMAT0003475
mmu-miR-374b-5p	AUAUAAUACAACCUGCUAAGUG	MIMAT0003727

*miRBase
